# Comparative genomic analysis of RNA-binding proteins across some drug resistant and sensitive *Staphylococcus aureus*

**DOI:** 10.1007/s00253-026-13906-x

**Published:** 2026-06-24

**Authors:** Magda M. Awad, Yehia A. Osman, Mohamed Abdelmoteleb

**Affiliations:** Botany Department, Faculty of Science, Mansoura University, Elgomhouria St., Mansoura, 35516 Egypt

**Keywords:** *Staphylococcus aureus*, RNA-binding protein, Drug-resistant, Genome, Virulence, Bioinformatics

## Abstract

The adaptability and persistence of *Staphylococcus aureus* in diverse host environments may be influenced by its genetic repertoire, including RNA-binding proteins (RBPs). These proteins play critical roles in regulating post-transcriptional processes; however, their significance in bacteria, particularly in pathogenic strains, remains poorly understood. This gap necessitates a comprehensive exploration of RBPs in bacterial pathogens such as *S. aureus* better to understand their potential contributions to virulence and adaptability. In this study, a bioinformatics-based analysis of the genomes and proteomes of 51 pathogenic *S. aureus* strains was conducted, focusing on their RNA-binding proteome (RBPome). The pathogenicity of all analyzed strains was confirmed, and both known and putative virulence genes were identified. In addition, strains were categorized as drug-resistant or drug-sensitive based on genomic data, providing insights into their genetic variation. Phylogenetic analysis based on single-nucleotide polymorphism (SNP) data revealed distinct groupings of major clonal complexes, highlighting evolutionary relationships among the strains. Eight conserved RNA-binding proteins were identified, and their RNA-binding domains and molecular dynamics were predicted. Notable differences in molecular phylogeny, RNA-binding domains, and dynamics were observed in cold shock proteins A and B and S1 RNA-binding domain proteins between resistant and sensitive strains. These findings suggest that RNA-binding proteins may play important roles in the pathogenicity and adaptability of *S. aureus* and could be potential molecular targets for future therapeutic strategies.

**Supplementary Information:**

The online version contains supplementary material available at 10.1007/s00253-026-13906-x.

## Introduction

*Staphylococcus aureus* is an opportunistic pathogen that asymptomatically colonizes the nasal cavity, skin, and mucosal membranes of humans and other mammals (Löffler et al. 2014; Cheung et al. 2021). Although often commensal, it can cause severe invasive infections associated with high morbidity and mortality. The emergence of multidrug-resistant strains, particularly methicillin-resistant *S. aureus* (MRSA), poses a major global health threat in both hospital and community settings and has been recognized by the World Health Organization as a priority pathogen (Bore et al. 2007; Saber et al. 2022). A key feature of *S. aureus*'s success is its ability to rapidly remodel its transcriptome in response to environmental stress, including antibiotic exposure and host immune pressure. This dynamic transcriptional reprogramming enables rapid adaptation through modulation of genes involved in cell wall synthesis, stress response, and virulence (Chabelskaya et al. 2010; Eyraud et al. 2014). While transcriptional control has been extensively studied, post-transcriptional regulation represents an additional critical layer governing bacterial adaptability.

RNA-binding proteins (RBPs) are emerging as central regulators of post-transcriptional gene expression in bacteria. By interacting with mRNAs and small regulatory RNAs (sRNAs), RBPs modulate mRNA stability, translation efficiency, and RNA–RNA interactions, thereby coordinating adaptive responses to environmental challenges (Quendera et al. 2020; Christopoulou and Granneman 2022; Menard et al. 2022). In *S. aureus*, sRNA-mediated regulation has been linked to both antibiotic resistance and virulence, including modulation of SpoVG, clumping factor B (ClfB), and delta hemolysin (Hld). These findings suggest that RBPs function as molecular switches influencing pathogenic phenotypes (Chabelskaya et al. 2010; Eyraud et al. 2014). Overall, RBPs serve as crucial molecular switches that enable *S. aureus* to survive hostile conditions, including antibiotic pressure and host immune responses. Understanding their precise mechanisms of action in antibiotic resistance and virulence regulation could provide novel insights into potential therapeutic targets for combating multidrug-resistant *S. aureus* infections (Morrison et al. 2012).

Although RNA-binding proteins (RBPs) have been extensively characterized in Gram-negative bacteria, their roles in Gram-positive organisms, including *Staphylococcus aureus*, remain comparatively underexplored (Van Assche et al. 2015). Well-studied RBPs such as Hfq and CsrA exhibit distinct distribution patterns and regulatory functions in Gram-positive species, suggesting fundamental differences in post-transcriptional control mechanisms (Christopoulou and Granneman 2022; Liao and Smirnov 2023). Furthermore, certain regulatory components, including RNase J and other small RNA-associated proteins, appear to be unique or functionally divergent in Gram-positive bacteria, indicating specialized RNA metabolism pathways (Guillet et al. 2013; Durand and Condon 2018; Rochat et al. 2018; Felden and Augagneur 2021; Brantl and Ul Haq 2023). This disparity highlights a critical knowledge gap in understanding post-transcriptional regulation in *S. aureus*, underscoring the need for systematic investigation of its RNA-binding proteome to clarify its role in stress adaptation, virulence, and antimicrobial resistance.

Unlike individual virulence or resistance genes that exert pathway-specific effects, RBPs act as global post-transcriptional regulators, modulating multiple downstream targets simultaneously. This systems-level regulatory capacity positions RBPs as potential higher-order modulators of bacterial adaptability. However, compared with Gram-negative bacteria, RBPs in Gram-positive pathogens such as *S. aureus* remain insufficiently characterized (Van Assche et al. 2015; Liao and Smirnov 2023). Investigating the RBPome, therefore, provides an opportunity to uncover coordinated regulatory mechanisms that may orchestrate resistance and virulence networks rather than focusing on isolated determinants.

Although numerous genes contribute to antimicrobial resistance and pathogenicity, variations in RNA-binding domains or structural dynamics of RBPs may exert cascading effects on multiple regulatory pathways. While bioinformatics analyses primarily identify associations rather than direct causality, comparative analysis between drug-resistant and drug-sensitive strains may generate mechanistic hypotheses regarding the regulatory role of RBPs in adaptive phenotypes (Christopoulou and Granneman 2022; Morrison et al. 2012). Experimental validation will be required to confirm these functional links.

In this study, two bioinformatics approaches were applied to investigate RNA-binding proteins (RBPs) in *S. aureus* strains. The first approach examined predicted multidrug resistance (MDR) and virulence genes and analyzed phylogenetic relationships among 51 strains, revealing correlations between evolutionary groupings and SNP-based patterns. The second approach involved comparative genomic and proteomic analyses of RBPs, identifying conserved RNA-binding domains and predicting potential molecular interactions involved in post-transcriptional regulation. We hypothesized that RBPs may be associated with bacterial adaptability, particularly with antibiotic resistance and virulence. Differences in RNA-binding domain composition and predicted interactions between drug-resistant and drug-sensitive strains were expected to provide insights into mechanisms of resistance and adaptation. These findings aim to improve understanding of RBPs in *S. aureus* pathogenicity and suggest potential targets for mitigating multidrug-resistant infections.

## Materials and methods

### Bacterial strains

#### Selection criteria

An initial literature search was performed using PubMed, Web of Science, and Google Scholar to identify *Staphylococcus aureus* strains previously classified as pathogenic or non-pathogenic, with documented drug-resistant or drug-sensitive phenotypes. Approximately 80 candidate strains were retrieved, ensuring representation of both resistant and susceptible isolates. To maximize dataset integrity, duplicate or highly similar genomes (29 strains) were excluded based on metadata, genomic similarity, and lineage redundancy, resulting in a final set of 51 representative *S. aureus* genomes (SupplementaryMaterial S1).

#### Genomic completeness and representativeness

Genomes and proteomes were obtained from the NCBI Genome database and the PubMLST database (Available at https://www.ncbi.nlm.nih.gov and https://pubmlst.org, Access date: January 2024) (Sayers et al. 2022; Jolley et al. 2018). Genome completeness and quality were assessed using Average Nucleotide Identity (ANI) analysis relative to *S. aureus* subsp. aureus N315 (Accession: BA000018.3) with the EZBioCloud ANI Calculator (Yoon et al. 2017). Both closed and high-quality draft genomes were included, while incomplete or low-quality assemblies were excluded. ANI analysis ensured accurate representation of genomic diversity, completeness, and phylogenetic distinction among selected strains.

The final dataset includes a representation of some drug-resistant (e.g., MRSA) and drug-sensitive strains. While not exhaustive of all global strains or resistance phenotypes, this dataset suggests hypothesis-generating comparisons of RNA-binding protein domains, virulence genes, and resistance-associated features. Consequently, all conclusions from these analyses are framed as preliminary insights, highlighting potential associations rather than definitive causal relationships.

### Pathogenicity, virulence, and resistance genes

Pathogenic potential of *S. aureus* strains was predicted using PathogenFinder 1.1 (Available at https://cge.food.dtu.dk/services/PathogenFinder/, Access date: Feb 2024) (Cosentino et al. 2013). Virulence genes were identified using VirulenceFinder 2.0 (Available at https://cge.food.dtu.dk/services/VirulenceFinder/, Access date: February 2024) (Malberg Tetzschner et al. 2020). Additionally, whole-genome sequences were screened against the Virulence Factor Database (VFDB) (Available at http://www.mgc.ac.cn/VFs/main.htm, Access date: February 2024) (Chen et al. 2016). Hits were considered significant at ≥ 60% gene coverage and ≥ 90% sequence identity (Joensen et al. 2014).

Acquired antimicrobial resistance genes were detected using ResFinder 4.6 (Available at http://cge.cbs.dtu.dk/services/, Access date: Feb 2024) (Bortolaia et al. 2020), which performs BLAST-based searches against a curated database of > 2000 resistance genes. Hits were considered significant at ≥ 90% sequence identity and ≥ 60% gene coverage, using the default BLAST E-value (10).

### Multi-locus sequence typing and phylogenetic analysis

Multi-locus sequence typing (MLST) of the selected *S. aureus* strains was performed using PubMLST (Available at https://pubmlst.org, Access date: March 2024). Clonal complexes (CCs) were assigned using BacWGSTdb 2.0 analysis (Available at http://bacdb.cn/BacWGSTdb/Tools.php, Access date: March 2024) **(**Ruan and Feng 2016). Minimum spanning trees were generated with *S. aureus* 08BA02176 (CP003808.1) as the reference genome and visualized using Phyloviz software (Available at https://online.phyloviz.net/index, Access date: March 2024) (Ribeiro-Gonçalves et al. 2016)..

Single-nucleotide polymorphisms (SNPs) among 51 complete genomes were identified using *S. aureus* subsp. aureus N315 (BA000018.3) as the reference genome. SNP calling and phylogenetic reconstruction were conducted with the online software package CSI Phylogeny (Available at https://cge.food.dtu.dk/services/CSIPhylogeny/, Access date: March 2024) following the concatenated alignment of high-quality SNPs (Kaas et al. 2014) based on concatenated high-quality SNP alignments. Filtering criteria included a minimum read depth of 10 ×, relative depth ≥ 10%, SNP quality score ≥ 30, mapping quality ≥ 25, *Z*-score ≥ 1.96, and a minimum inter-SNP distance of 10 bp to reduce recombination bias. Phylogenetic trees were constructed using 1000 bootstrap replicates and visualized with iTOL (Available at https://itol.embl.de/, Access date: March 2024) (Letunic and Bork 2007).

### Proteome-wide survey (PWS) of RNA-binding proteins

A proteome-wide survey (PWS) was conducted to identify, quantify, and analyze RNA-binding protein domains (RBPDs) across 51 *Staphylococcus aureus* strains, aiming to elucidate their roles in gene regulation, virulence, pathogenesis, and antibiotic resistance. Computational domain predictions were performed across complete proteomes based on sequence and structural features (Ghosh and Sowdhamini 2017).

Protein family annotation and conserved RNA-binding motif identification were carried out using Protein Families Database (Pfam version 36.0, http://pfam.xfam.org/) (Mistry et al. 2021). Conserved domains were further validated using the Conserved Domain Database (CDD version 3.21, https://www.ncbi.nlm.nih.gov/Structure/bwrpsb/bwrpsb.cgi) (Wang et al. 2023). Motif verification was performed with MOTIF Search (https://www.genome.jp/tools/motif/), and integrated domain confirmation was verified through InterPro database (InterPro 100.0, https://www.ebi.ac.uk/interpro/search/sequence/) (Ghosh and Sowdhamini 2017). All databases were accessed in April 2024.

The RBPD dataset was structured with strains as columns and RNA-binding domains as rows, where each cell represented the domain count per strain (SupplementaryMaterial S2). Descriptive statistics (mean ± SD) were calculated for each domain across strains. One-way ANOVA was conducted separately for each domain using the R program version 4.4.1 (Available at https://cran.r-project.org/bin/windows/base/, Access date: April 2024) to assess differences in domain distribution. Statistical significance was set at *p* < 0.05, while values between 0.05 and 0.1 were reported as suggestive but not significant (Wasserstein and Lazar 2016; Cesana 2018; Di Leo and Sardanelli 2020; White et al. 2022). Tukey’s Honestly Significant Difference (HSD) test was applied for post hoc pairwise comparisons (Ghosh and Sowdhamini 2016).

Additionally, Analysis of Covariance (ANCOVA) was performed to examine associations between RBPD counts and virulence genes, as well as RBPD counts and antimicrobial resistance genes. Effect size difference plots were generated in R (Peng 2016) to visualize RBPD distribution and highlight significant correlations with virulence and multidrug resistance determinants (Jamieson 2004).

### Literature mining, RBP identification, and proteome-wide screening

A literature parsing step was conducted to investigate RNA-binding proteins (RBPs) in *S. aureus* using PubMed, Web of Science, and Google Scholar. Searches were performed using the query “RNA-binding protein AND *S. aureus*,” and results were filtered to identify RBPs specifically associated with virulence and pathogenicity. Previously reported RBPs of *Staphylococcus* spp. were retrieved from the NCBI protein database (Ghosh and Sowdhamini 2016, 2017), and a local RBP database was constructed for downstream analyses.

Proteomes of 51 *S. aureus* strains were compared against the local RBP database using BLASTP with an E-value cutoff of 10⁻^5^ to ensure statistical significance (Altschul et al. 1990). Identified hits were clustered using a sequence identity threshold of ≥ 30% and query coverage ≥ 70%. A 30% identity cutoff is commonly considered the lower boundary for homologous inference (Söding 2005), while ≥ 70% coverage ensures substantial alignment and excludes short, non-representative fragments. These parameters balance sensitivity and specificity and are consistent with comparative genomics standards (Kaushik et al. 2013).

Multiple sequence alignments of selected RBPs were performed using COBALT (available at https://www.ncbi.nlm.nih.gov/tools/cobalt/re_cobalt.cgi, Access date: June 2024) (Edgar 2004).

Proteome-wide screening (PWS) was conducted to quantify eight RBPs (CspA, CspB, CvfB, Hfq, RsmH, RsmI, S1_RBP, and SpoVG) and their associated domains across the 51 strains. Protein sequences were queried against Pfam (v36.0) (Mistry et al. 2021) to identify conserved RNA-binding domains and characterize domain architectures. RBP abundance was visualized using GraphPad Prism (Available at https://www.graphpad.com/, Access date: May 2024). Identified Pfam domains were further annotated and visualized using TBtools a genomic and proteomic analysis software (Available at https://apps.microsoft.com/detail/9nvr9lb426p2?hl=en-US&gl=US, Access date: May 2024) (Chen et al. 2020), enabling detailed representation of conserved motifs and functional regions.

### Molecular dynamics simulation of RBPs

To compare the dynamic behavior of three RNA-binding proteins (CspA, CspB, and S1_RBP) harboring sequence variations between drug-resistant and drug-sensitive *S. aureus* strains, molecular dynamics simulations (MDS) were performed to evaluate protein stability and conformational changes in a simulated biological environment.

As experimental 3D structures were not available in the Protein Data Bank, protein models were predicted using AlphaFold (v1.8) (Ruff and Pappu 2021) and visualized with PyMOL (DeLano 2002; Yuan et al. 2016). Molecular dynamics simulations were conducted using GROMACS 2023.5 (GNU, general public license; http://www.gromacs.org) following this tutorial (http://www.mdtutorials.com/gmx/lysozyme/index.html) under the CHARMM36 force field, with protein topology generated via the *pdb2gmx* module (Vanommeslaeghe et al. 2010; Abraham et al. 2015); Simulations were carried out for 100 ns following standard MD protocols.

Protein stability and structural behavior were assessed using backbone root mean square deviation (RMSD), which measures deviation from the initial predicted structure and reflects overall conformational stability (Dixit et al. 2006). Root mean square fluctuation (RMSF) analysis was performed to evaluate residue-level flexibility and structural domain mobility (Martínez 2015). The radius of gyration (RoG) was calculated to assess structural compactness throughout the simulation trajectory (Baig et al. 2014). All plots were generated were generated using ggplot2 in R., the code is available online on this GitHub repository (https://github.com/ttobio/MDS), and clustering of MD trajectories was performed using ttclust (Tubiana et al. 2018) to identify the most populated conformational cluster. The representative structure from the largest cluster was extracted and compared with the initial model using PyMOL (DeLano 2002; Yuan et al. 2016).

## Results

### Pathogenicity and virulence and resistance genes

**Fig. 1:**
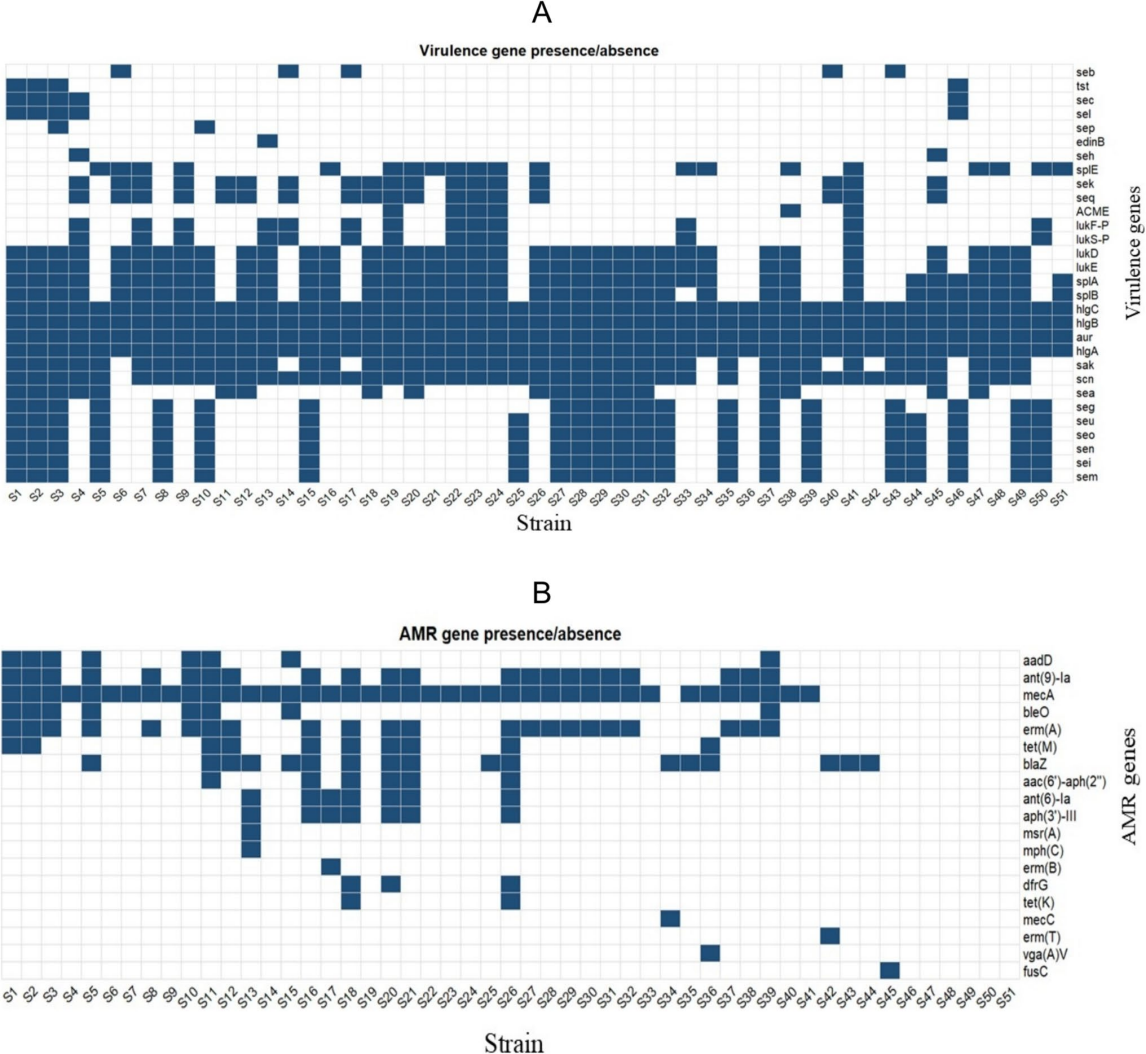
**A** Heatmap showing the distribution (presence/absence) of virulence genes in different strains (numbered S1- S51). **B** Heatmap showing the distribution of antibiotic resistance genes. (Dark blue: present; White: Absent)

All tested *S. aureus* strains were identified as human pathogens. Using VirulenceFinder and VFDB, a total of 30 virulence-related genes were identified in the genomes of the tested *S. aureus* strains. Figure 1A illustrates the distribution (presence/absence) of identified virulence genes per isolate. All tested strains carried the *hlgA, hlgB,* and *hlgC* genes, which are responsible for gamma-hemolysin chain II precursor, gamma-hemolysin component B precursor, and gamma-hemolysin component C, respectively. A unique presence of *edinB*, responsible for epidermal cell differentiation inhibitor B, was reported in strain S13 (CP003194.1). The largest number of virulence genes was in S3, while the smallest number was in S36 (Supplementary Material S3).

Antibiotic resistance was predicted based on the presence of resistance genes in each strain. Strains were classified as multi-drug-resistant (MDR) if they carried genes conferring resistance to one or more antibiotics, while strains lacking detectable resistance genes were considered drug-sensitive. Out of the 51 tested *S. aureus* strains, 45 were predicted to exhibit resistance to at least one antibiotic, with mecA detected in 44 strains, indicating methicillin resistance, and blaZ detected in 17 strains, conferring β-lactamase-mediated resistance. The fusc gene, responsible for fusidic acid resistance, was predicted only in strain S45. The remaining six strains (S46–S51) lacked detectable resistance genes and were classified as drug-sensitive. The predicted distribution of antibiotic resistance across all strains is shown in Fig. 1B and Supplementary Material S4.

### Multi-locus sequence typing and phylogenetic analysis

MLST typing of *S. aureus* genomes identified 22 distinct sequence types (STs) and eight clonal complexes (CCs). The most frequent sequence types were ST8 (*n* = 10) and ST239 (*n* = 7). Strains S13, S14, S15, S17, S40, S43, S44, and S46 could not be assigned to any clonal complex and are listed as NI (Non-identified) in Table 1. Most USA strains in this study belonged to CC5 or CC8.

**Table 1:** Sequence types and clonal complexes of *S. aureus* strains

Strain	Clonal complex (CC)	ST	*arc*	*aroe*	*glpf*	*gmk*	*pta*	*tpi*	*yqil*
S29	CC5	228	1	4	1	4	12	24	29
S30	CC5	228	1	4	1	4	12	24	29
S31	CC5	228	1	4	1	4	12	24	29
S28	CC5	228	1	4	1	4	12	24	29
S37	CC5	228	1	4	1	4	12	24	29
S27	CC5	228	1	4	1	4	12	24	29
S49	CC5	5	1	4	1	4	12	1	10
S3	CC5	5	1	4	1	4	12	1	10
S2	CC5	5	1	4	1	4	12	1	10
S1	CC5	5	1	4	1	4	12	1	10
S10	CC5	225	1	4	1	4	12	25	10
S8	CC22	105	1	4	1	4	12	1	28
S32	CC22	105	1	4	1	4	12	1	28
S43	NI	27	3	3	1	1	1	22	10
S15	NI	72	1	4	1	8	4	4	3
S13	NI	80	1	3	1	14	11	51	10
S4	CC1	1	1	1	1	1	1	1	1
S45	CC1	1	1	1	1	1	1	1	1
S25	CC22	22	7	6	1	5	8	8	6
S35	CC22	22	7	6	1	5	8	8	6
S39	CC45	45	10	14	8	6	10	3	2
S5	CC30	36	2	2	2	2	3	3	2
S50	CC30	243	2	2	5	2	6	3	2
S42	CC15	398	3	35	19	2	20	26	39
S36	CC15	398	3	35	19	2	20	26	39
S33	CC93	93	6	64	44	2	43	55	51
S46	NI	151	6	72	12	43	49	67	59
S34	CC93	425	18	33	6	20	7	50	48
S44	NI	50	16	16	12	2	13	13	15
S40	NI	59	19	23	15	2	19	20	15
S14	NI	59	19	23	15	2	19	20	15
S17	NI	59	19	23	15	2	19	20	15
S11	CC8	239	2	3	1	1	4	4	3
S12	CC8	239	2	3	1	1	4	4	3
S16	CC8	239	2	3	1	1	4	4	3
S21	CC8	239	2	3	1	1	4	4	3
S20	CC8	239	2	3	1	1	4	4	3
S18	CC8	239	2	3	1	1	4	4	3
S26	CC8	239	2	3	1	1	4	4	3
S51	CC8	8	3	3	1	1	4	4	3
S48	CC8	8	3	3	1	1	4	4	3
S6	CC8	250	3	3	1	1	4	4	16
S47	CC8	254	3	32	1	1	4	4	3
S38	CC8	8	3	3	1	1	4	4	3
S41	CC8	8	3	3	1	1	4	4	3
S9	CC8	8	3	3	1	1	4	4	3
S19	CC8	8	3	3	1	1	4	4	3
S7	CC8	8	3	3	1	1	4	4	3
S23	CC8	8	3	3	1	1	4	4	3
S24	CC8	8	3	3	1	1	4	4	3
S22	CC8	8	3	3	1	1	4	4	3

*NI* non-identified clonal complex, *CC* clonal complexes, *ST* sequence type, *arc* arginine catabolic pathway gene, *aroE* shikimate dehydrogenase gene, which is involved in the biosynthesis of aromatic amino acids,* glpF* glycerol uptake facilitator protein gene, *gmk* guanylate kinase gene, crucial for nucleotide metabolism, *pta* phosphate acetyltransferase gene, associated with acetate metabolism, *tpi* triosephosphate isomerase gene, a key enzyme in glycolysis and gluconeogenesis, *yqiL* putative gene, often referenced in *S.* *aureus* studies, potentially involved in regulatory or unknown functions

**Fig. 2:**
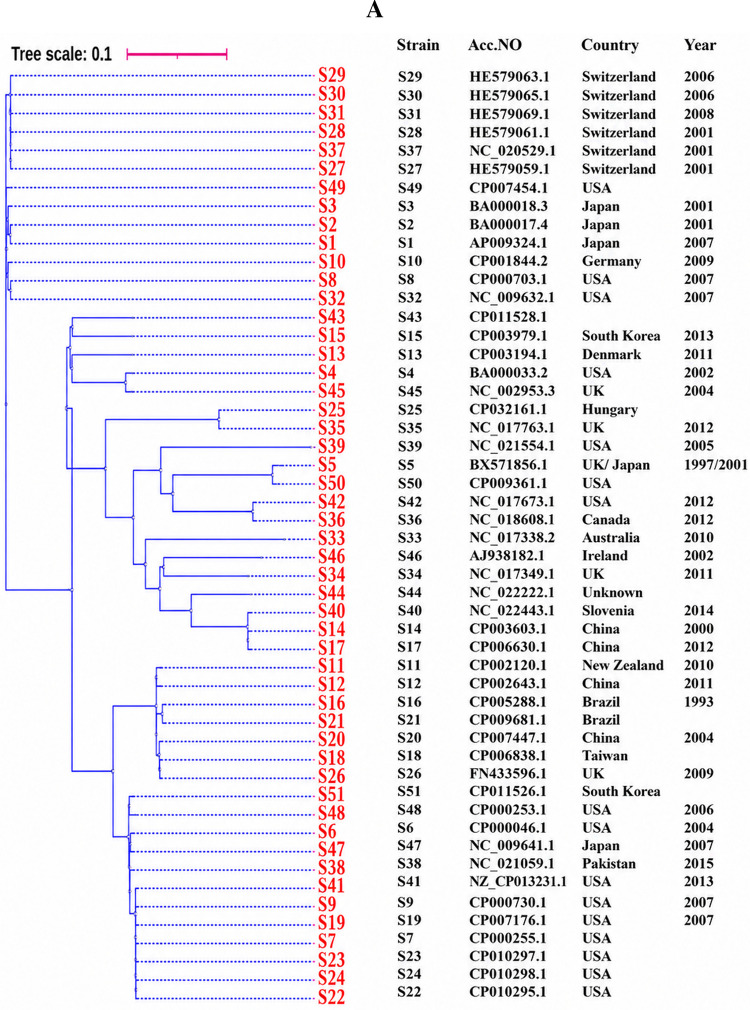

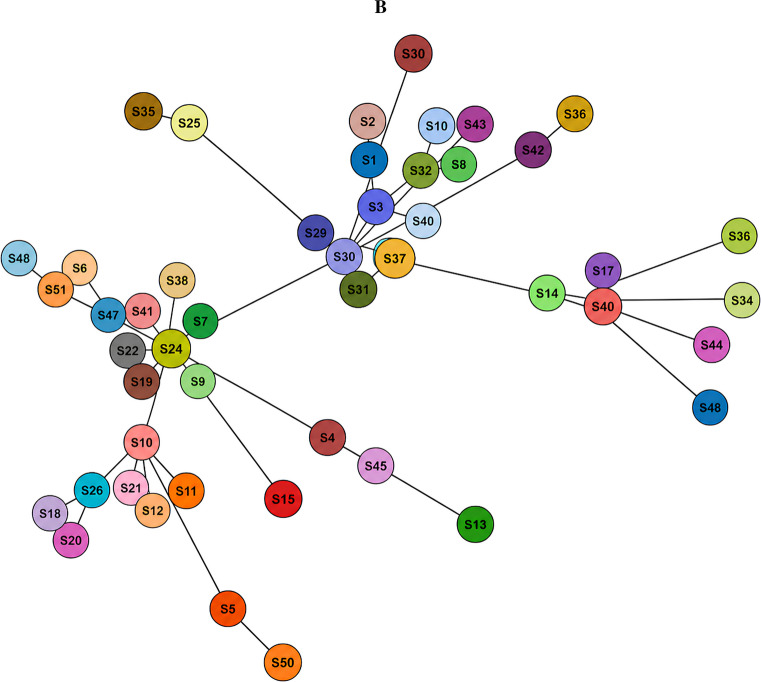
**A** SNP-based phylogenetic tree of *S. aureus* genomes. **B** A minimum-spanning tree based on the whole-genome multilocus sequence typing profiles of *S. aureus* strains. Branch labels represent the allelic distances.

A SNP-based phylogenetic tree was constructed using *S. aureus* subsp. *aureus* N315 (Accession No. BA000018.3) as the reference. Strains clustered according to their clonal complex or sequence type. Additionally, a minimum spanning tree (MST) was generated using *S. aureus* 08BA02176 (CP003808.1) as the reference genome. MST analysis of 51 strains revealed distinct genetic clusters, with closely related strains forming tight groups and more divergent isolates showing longer branch separations. The central cluster contained strains with shared genetic features, suggesting common ancestry, while peripheral nodes represented more genetically distinct isolates from independent evolutionary events. Multidrug-resistant (MDR) strains were spread across multiple branches, indicating that resistance arose independently in different lineages. Overall, these analyses highlight the genetic diversity and heterogeneity of *S. aureus*, which may contribute to differences in virulence and drug resistance (Fig. 2A and B).

### Proteome-wide survey (PWS) of RNA-binding proteins

**Fig. 3:**
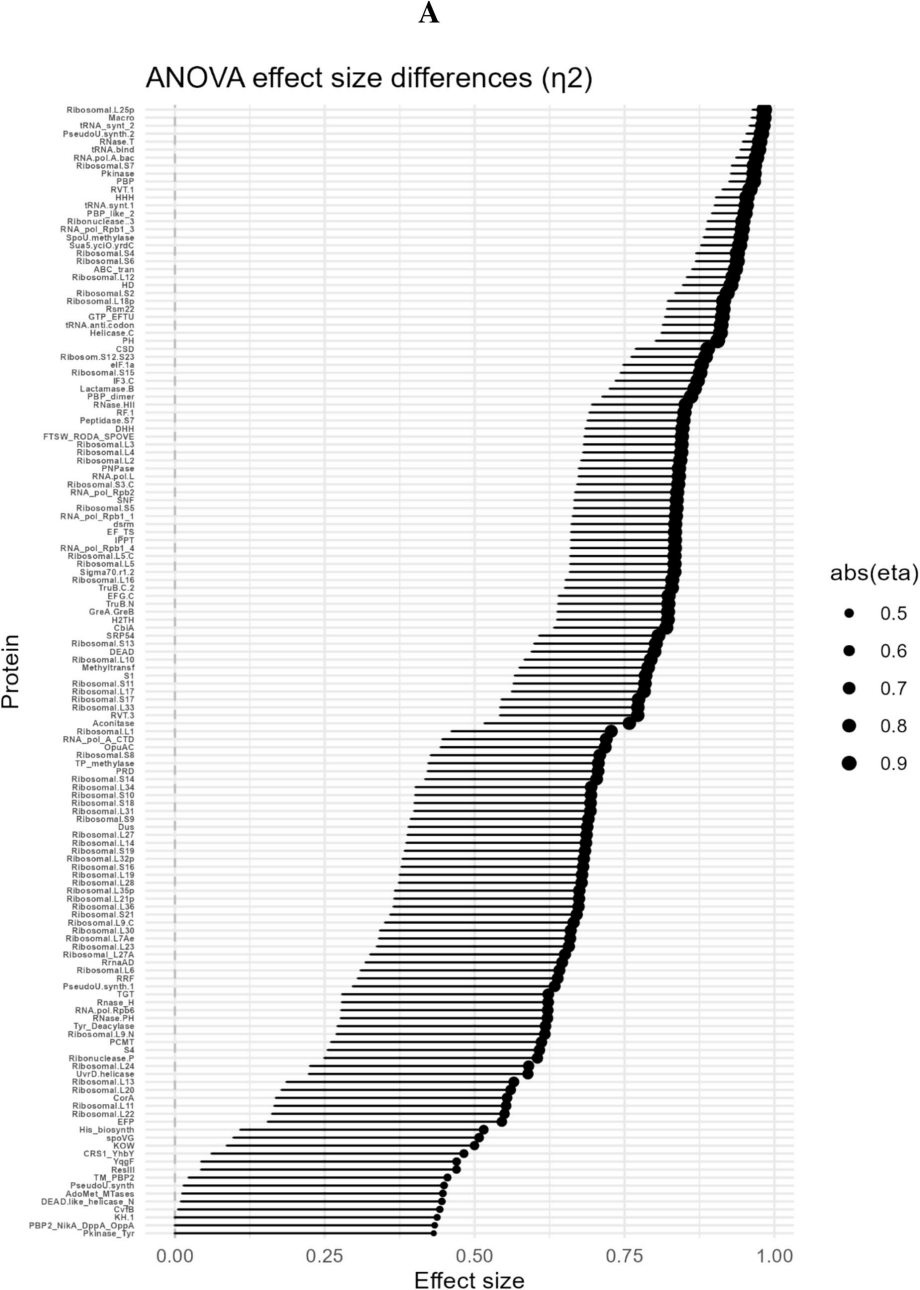

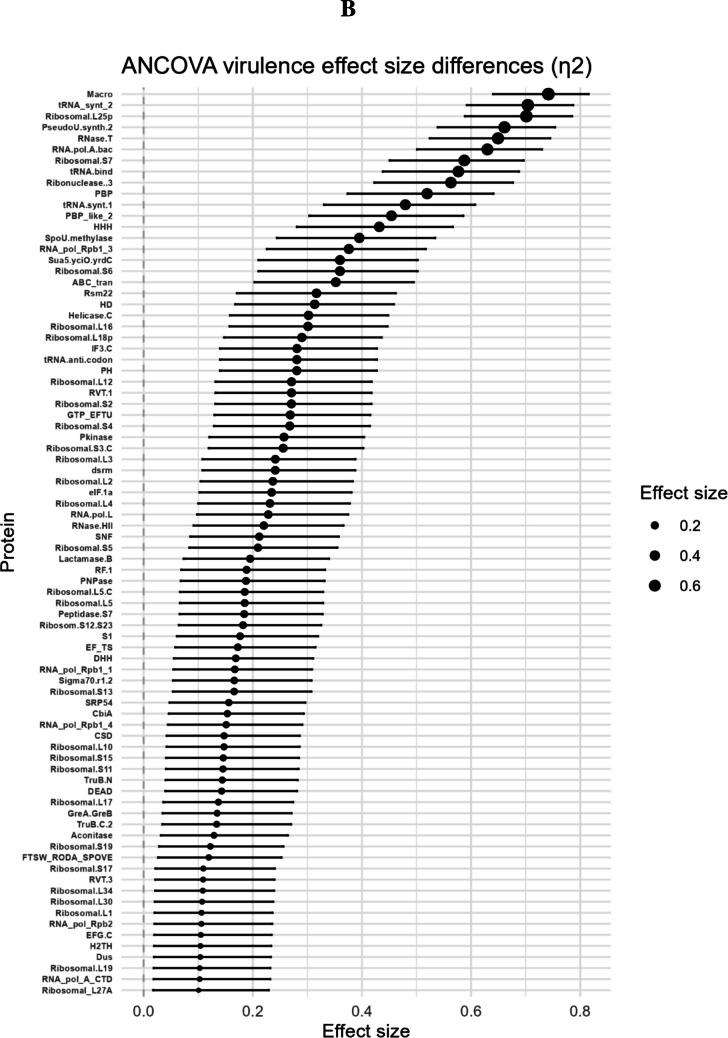
**A** ANOVA and (**B**) ANCOVA analyses between RBPD and virulence genes

Proteome-wide screening of the studied *S. aureus* strains identified 166 Pfam RNA-binding domains (RBDs), with the ABC transporter domain being the most common. Multi-drug-resistant strains generally had larger proteomes, with strain S37 having the largest. ANOVA analysis showed that HIT, Hfq, LSM, and RGS domains did not vary significantly, while other RBPDs exhibited notable differences among strains (Supplementary Material S5). Effect sizes (*η*^2^) highlighted domains with the most pronounced variation, emphasizing differences in RBPD distribution across the analyzed strains (Fig. 3A).

ANCOVA analysis of 166 RNA-binding protein domains (RBPDs) revealed that 82 significantly influenced *S. aureus* virulence. Most domains had small effects, but Macro, tRNA_synt_2, Ribosomal L25p, and PseudoU_synth showed larger effect sizes, which may indicate a stronger role in virulence regulation (Fig. 3B). Ribosomal and metabolic RBPDs that positively correlated with virulence are listed in (Supplementary Material S6), suggesting their potential contribution to pathogenesis.

**Fig. 4:**
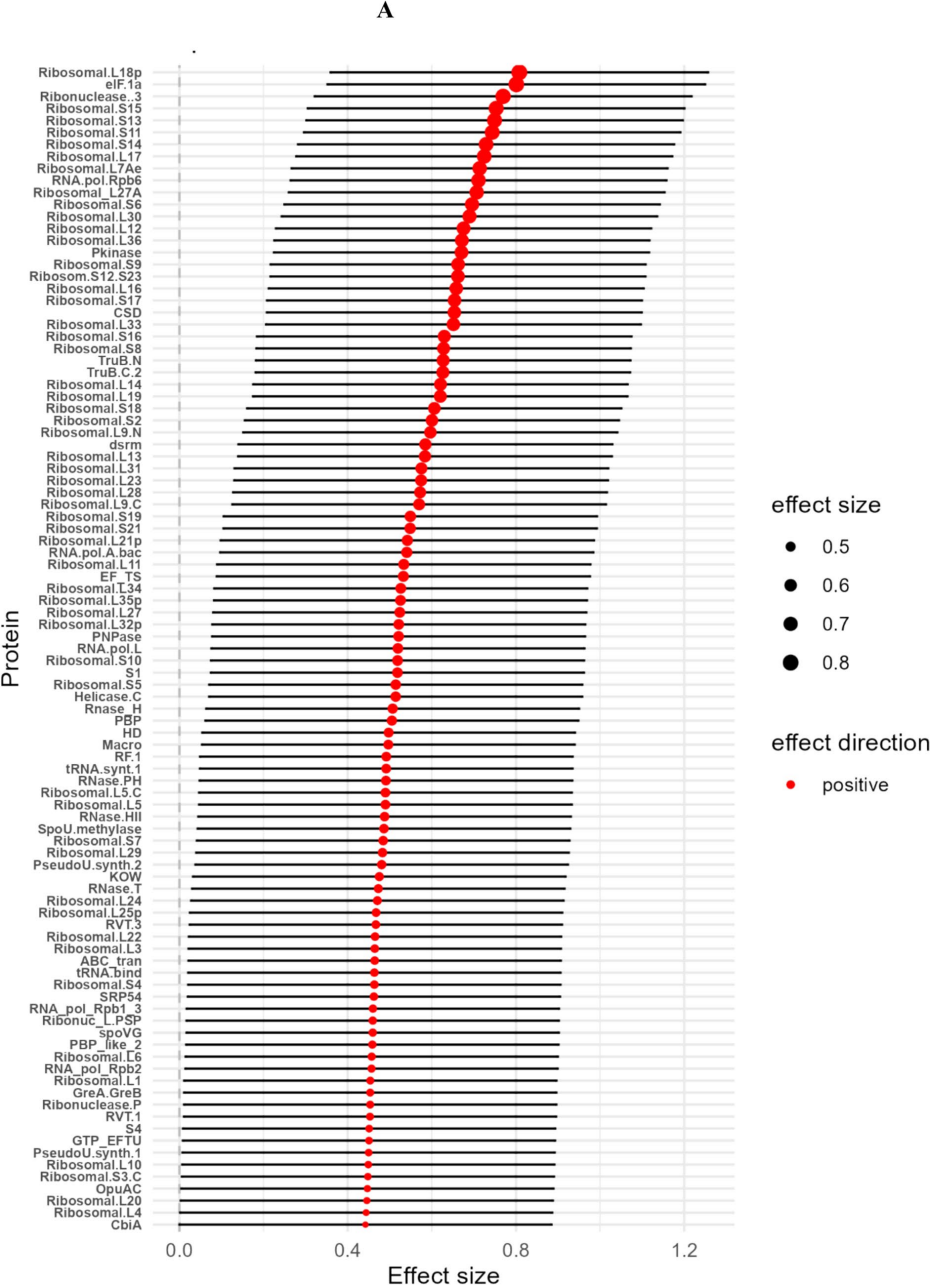

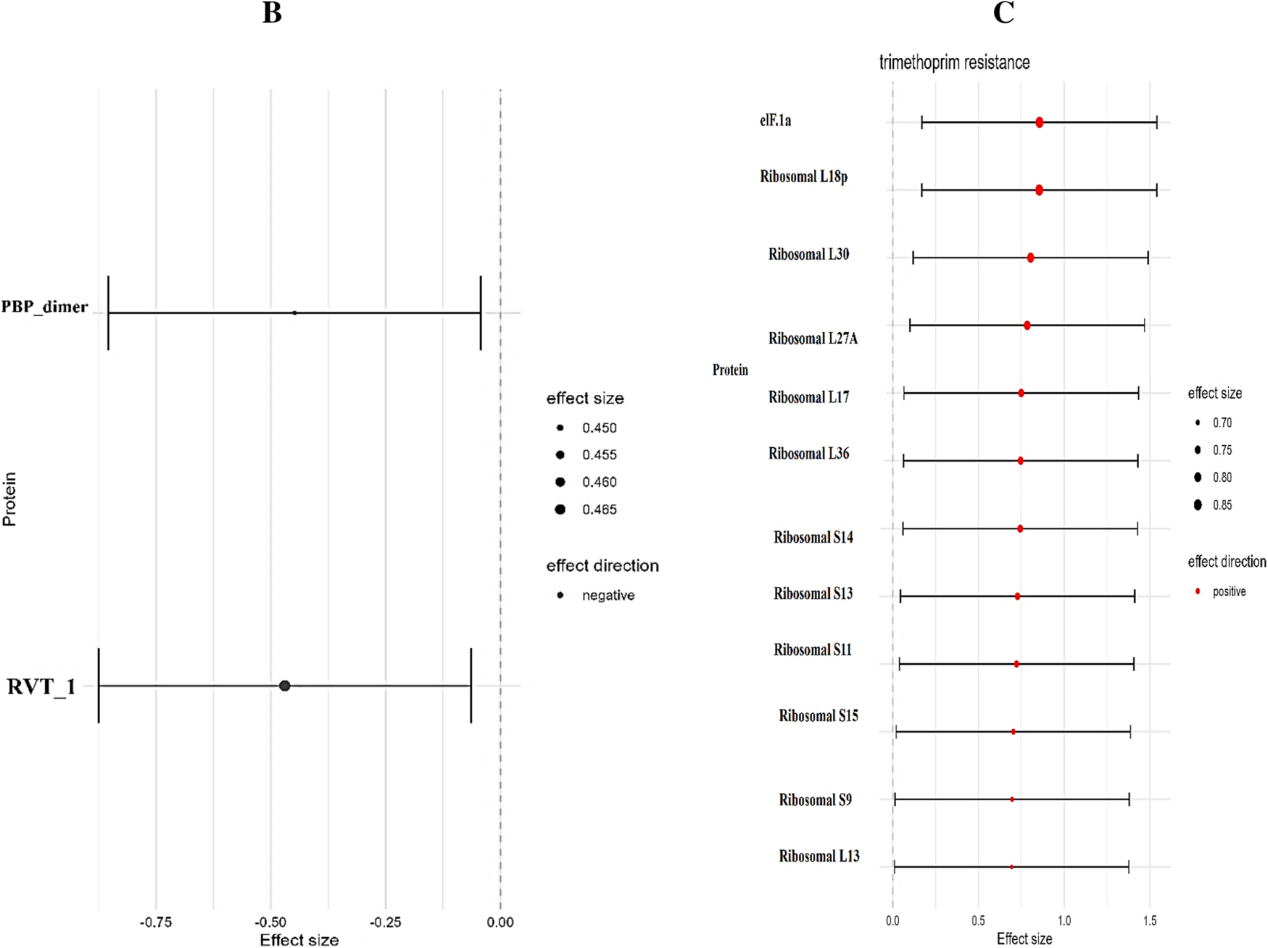

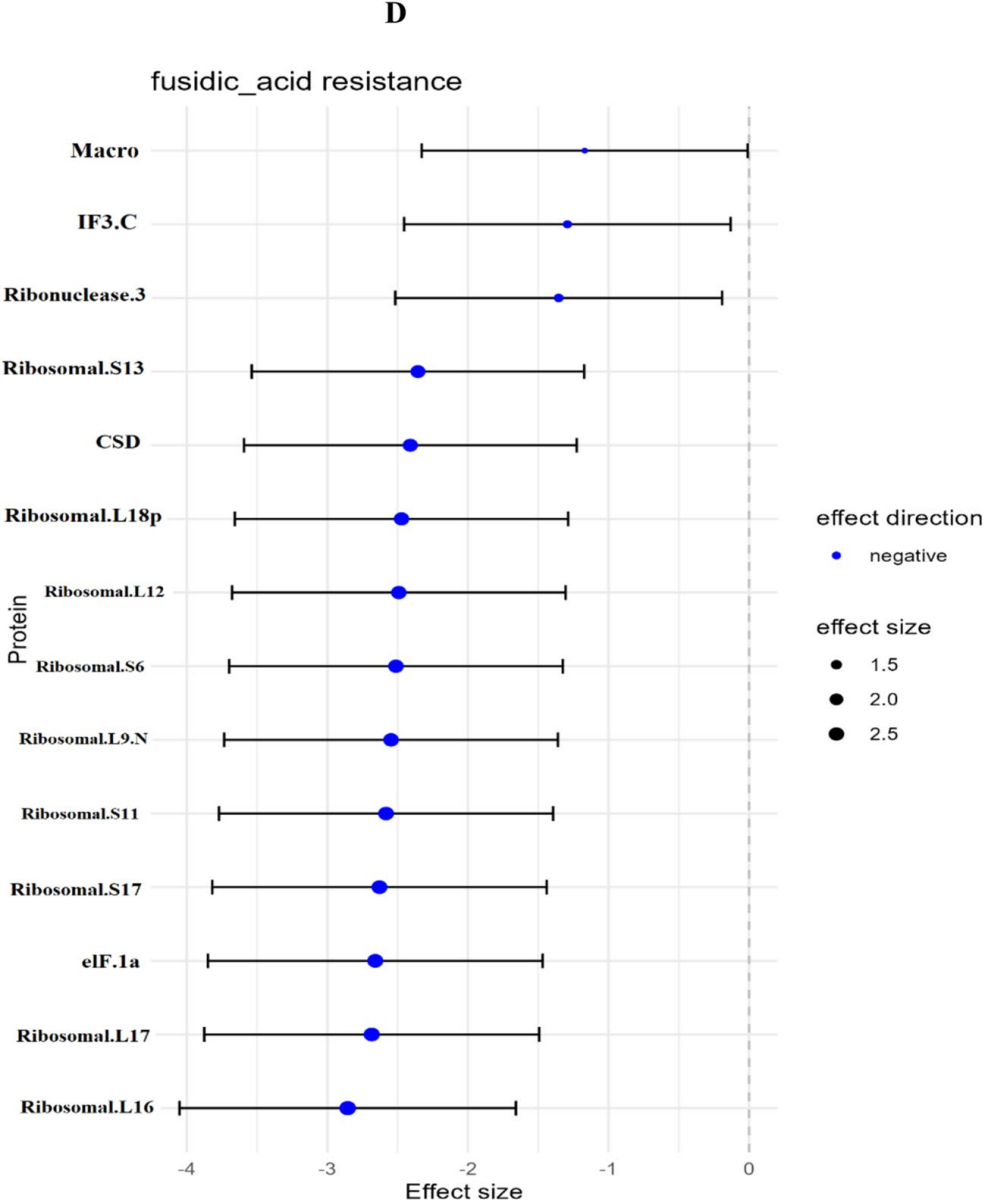
ANCOVA effect size plots illustrating the impact of RNA-binding protein domains (RBPDs) on antibiotic resistance. The *x*-axis represents the effect size, while the *y*-axis lists the RBPDs. **A** Aminoglycoside resistance. **B** Beta-lactam and other antibiotic resistance. **C** Trimethoprim resistance. **D** Fusidic acid resistance

ANCOVA analysis and effect size difference plots were used to examine the relationship between RNA-binding protein domains (RBPDs) and antibiotic resistance in *S. aureus*. The analysis was divided into four categories, each showing correlations between RBPDs and specific antibiotics. In Fig. 4A, correlations with aminoglycoside resistance (e.g., amikacin, gentamicin, kanamycin, tobramycin, and others) are presented, with the *x*-axis showing correlation coefficients (0–1.0) and the *y*-axis listing RBPDs. Approximately 121 RBPDs displayed positive correlations (red spots), and nine RBPDs showed particularly strong associations, mainly involving ribosomal proteins, transcription, and RNA metabolism (Supplementary Material S7). These findings suggest that specific RBPDs may contribute to aminoglycoside resistance in *S. aureus*, warranting further investigation into their precise roles and interactions.

The second category assessed the impact of RBPDs on *S. aureus* resistance to multiple antibiotics, including beta-lactams (e.g., amoxicillin-clavulanic acid, cefepime, meropenem), pleuromutilins (tiamulin), and streptogramin A antibiotics (dalfopristin, quinupristin-dalfopristin). The analysis (Fig. 4B) focused on two RBPDs, PBP-dimer and RVT1, with effect sizes shown on the x-axis and proteins on the y-axis. Both proteins were significantly associated with increased susceptibility to amoxicillin-clavulanic acid (*p* = *0.00036* for PBP-dimer; *p* = *2.90* × *10⁻*^*5*^ for RVT1). However, overlapping confidence intervals indicate that the difference between their effects was not statistically significant, suggesting these RBPDs may enhance susceptibility rather than confer resistance.

The third category explored predicted associations between RBPDs and *S. aureus* resistance to trimethoprim (Fig. 4C). Twelve RBPDs including elF.1a, Ribosomal L18p, L30, L27A, L17, L36, S14, S13, S11, S15, S9, and L13 were evaluated. Among these, Ribosomal L17, L27A, and L30 were predicted to have the strongest influence on trimethoprim resistance, suggesting a substantial role in modulating resistance mechanisms. Clustering of ribosomal proteins implies potential shared functional pathways, highlighting the ribosome as a key target in resistance prediction.

The fourth category assessed predicted effects of RBPDs on fusidic acid susceptibility (Fig. 4D). Domains analyzed included Macro, IF3C, Ribonuclease 3, several ribosomal proteins (e.g., L12, L16, L17, L18p, L9N, S6, S11, S13, S17), CSD, and eIF.1a. All domains displayed negative predicted effect sizes, suggesting these RBPDs may increase susceptibility rather than confer resistance. Clustering patterns indicate shared mechanisms among ribosomal subunits and elongation factors, with proteins such as IF3C predicted to play a notable role, potentially by enhancing ribosome recycling or preventing premature termination, thereby mitigating fusidic acid activity.

### Comparison of RNA-binding proteins across strains

A literature-based analysis retrieved approximately 784 PubMed, 200 Web of Science, and 15,100 Google Scholar results. From these, eight RBPs CspA, CspB, CvfB, Hfq, RsmH, RsmI, S1, and SpoVG were identified as key regulators of *S. aureus* pathogenesis (Supplementary Material S8). Their prioritization was based on functional relevance, frequency in the literature, and predicted roles in bacterial survival, stress response, and adaptation, suggesting these RBPs may serve as critical modulators of virulence.

### Multiple sequence alignments and phylogenetic analyses

Multiple sequence alignment (MSA) of the eight RNA-binding proteins (RBPs) across 51 *S. aureus* strains revealed highly conserved regions important for pathogenicity and virulence. CvfB, Hfq, RsmH, RsmI, and SpoVG showed minimal variation among strains, with strong conservation in dynamic hotspot regions and RNA-interacting sites, indicating evolutionary pressure to maintain their sequences and structures. In contrast, CspA, CspB, and S1_RBP displayed notable sequence variations, particularly in strains selected for multidrug resistance and high virulence (Supplementary Materials S9 and 10).

### Conserved domains of RNA-binding proteins

**Fig. 5:**
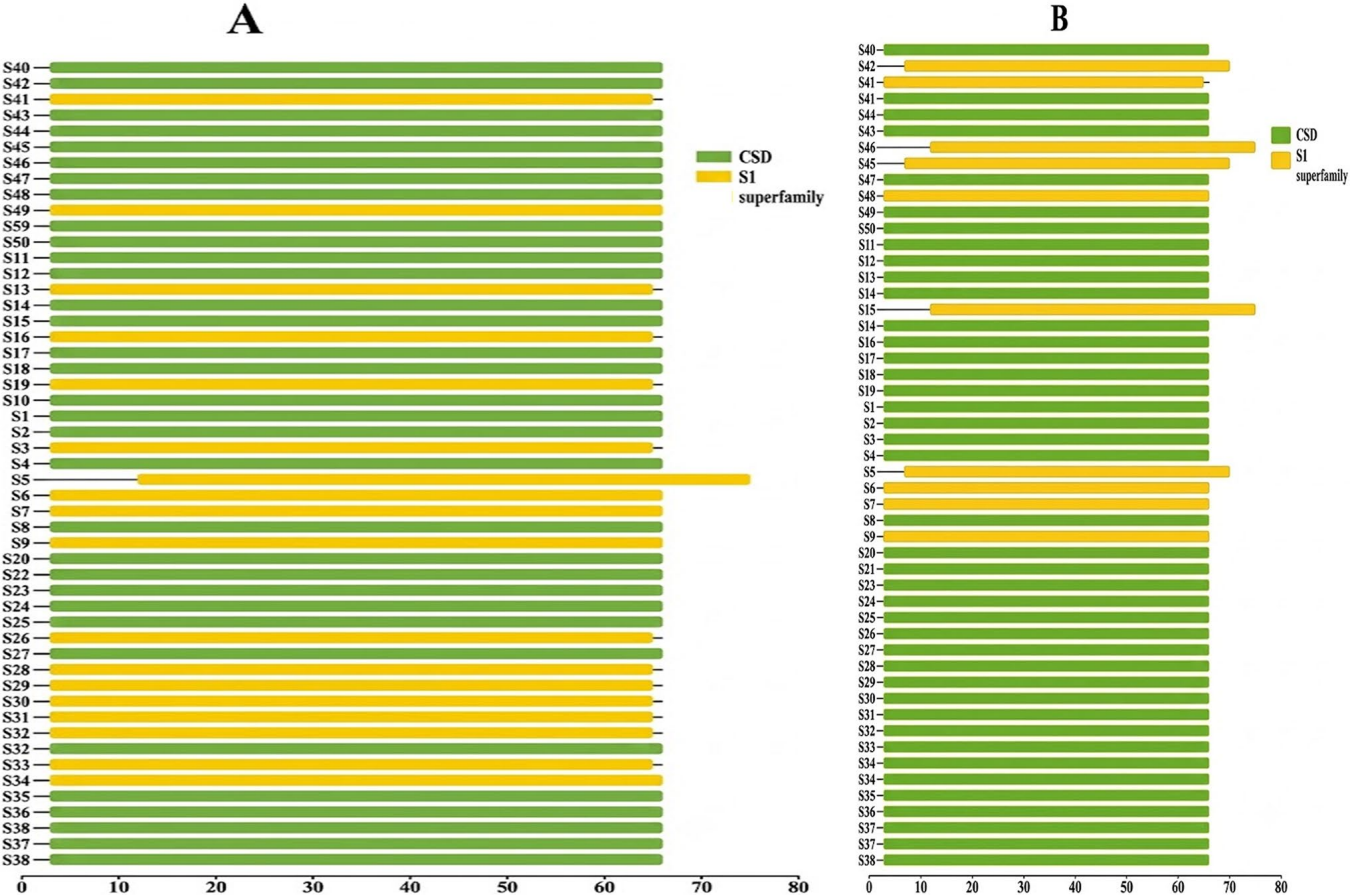

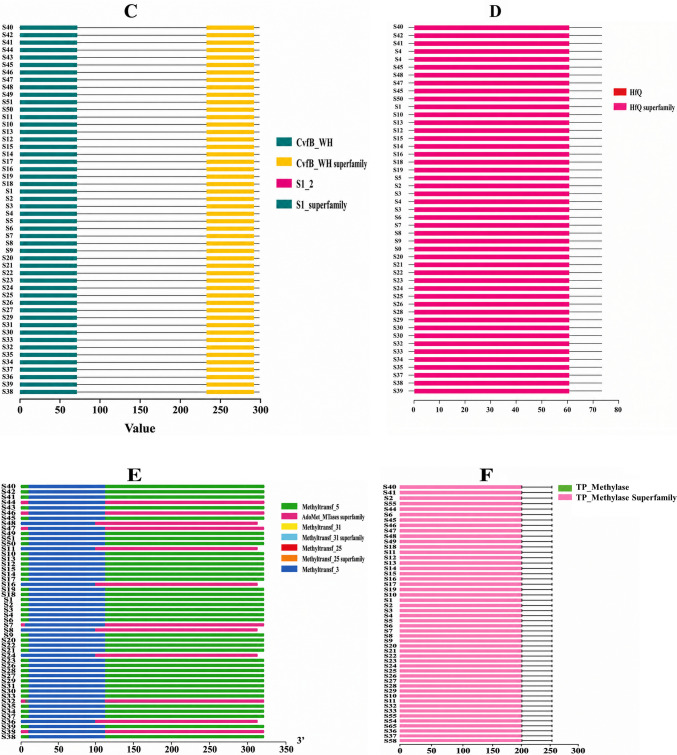

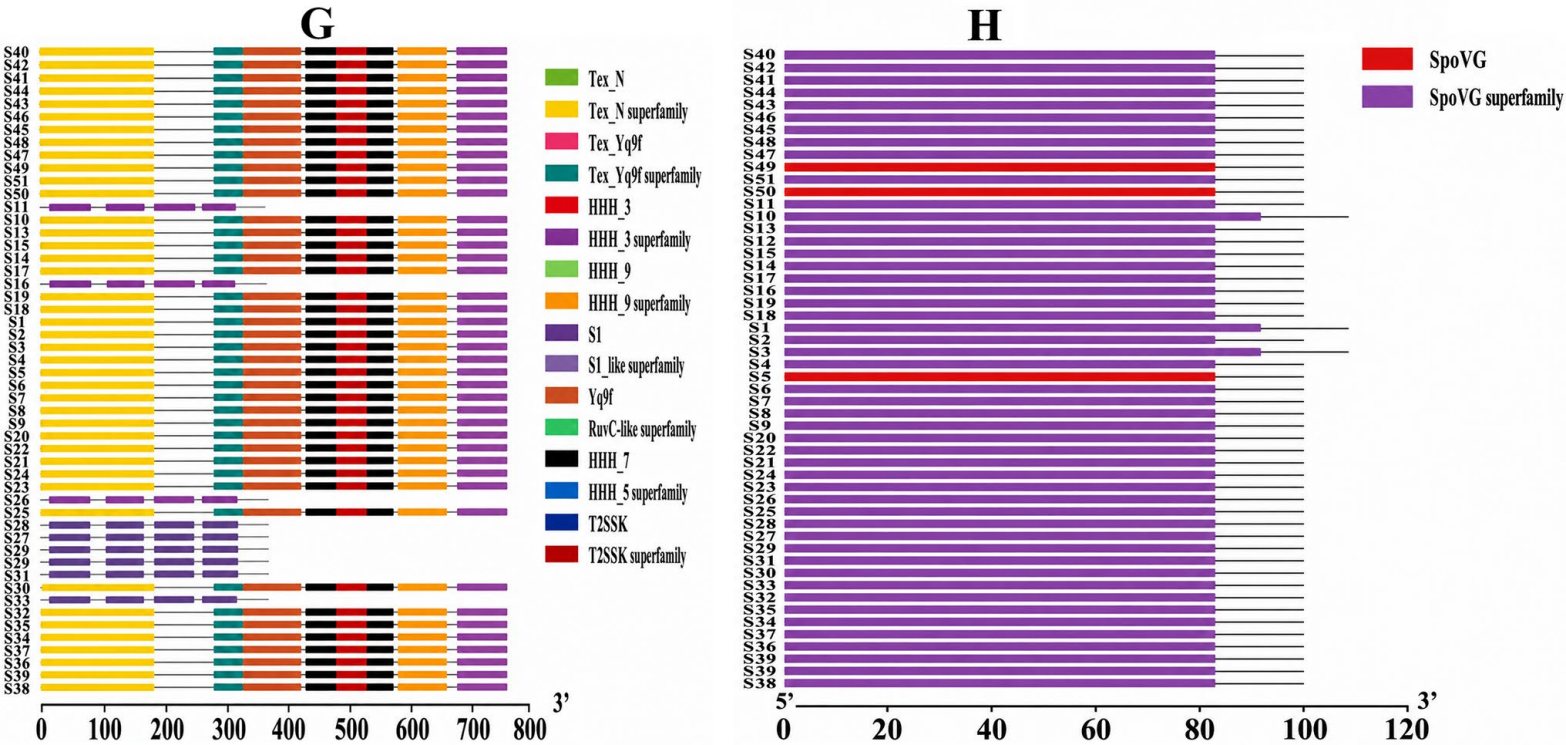
Composition of conserved domains in eight RBPs across the 51 *S. aureus* strains. **A** CspA. **B** CspB. **C** CvfB. **D** Hfq. **E** RsmH. **F** RsmI. **G** S1_RBP. **H** SpoVG

Proteome-wide screening (PWS) quantified RNA-binding protein domains (RBPDs) for eight key RBPs across 51 *S. aureus* strains (Supplementary Material S11). The frequency and distribution of RBPDs, visualized in Supplementary Material S12, revealed both highly conserved domains and strain-specific variations. Pfam domain analysis further identified conserved RNA-binding motifs, and detailed domain architecture, examined with TBtools (Fig. 5) highlighted structural differences that could underlie strain-specific regulatory mechanisms. The results suggest the evolutionary conservation of critical RNA-binding functions alongside variations that may influence *S. aureus* pathogenicity and adaptability.

### Molecular dynamics simulation of RBPs

**Fig. 6:**
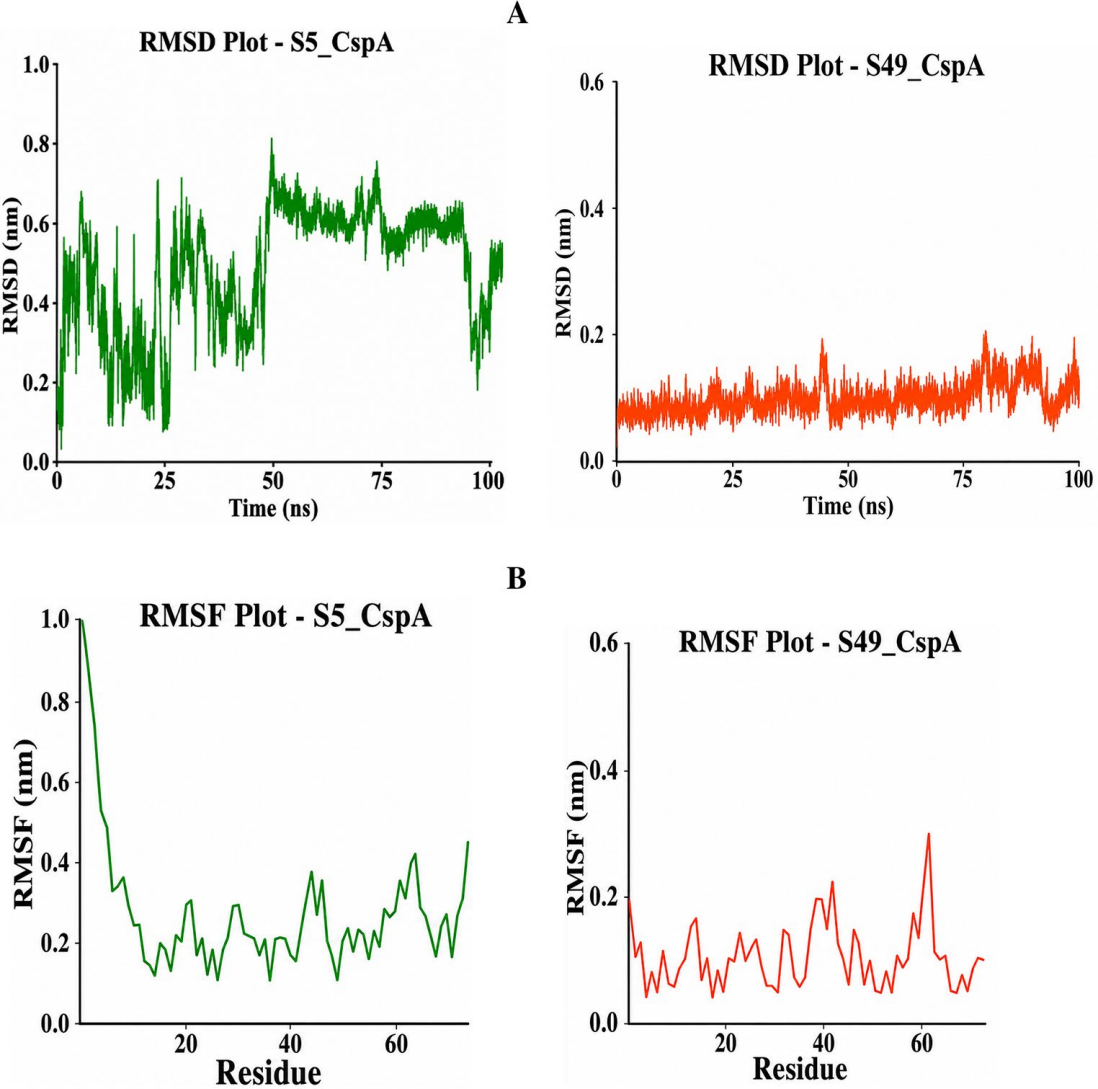

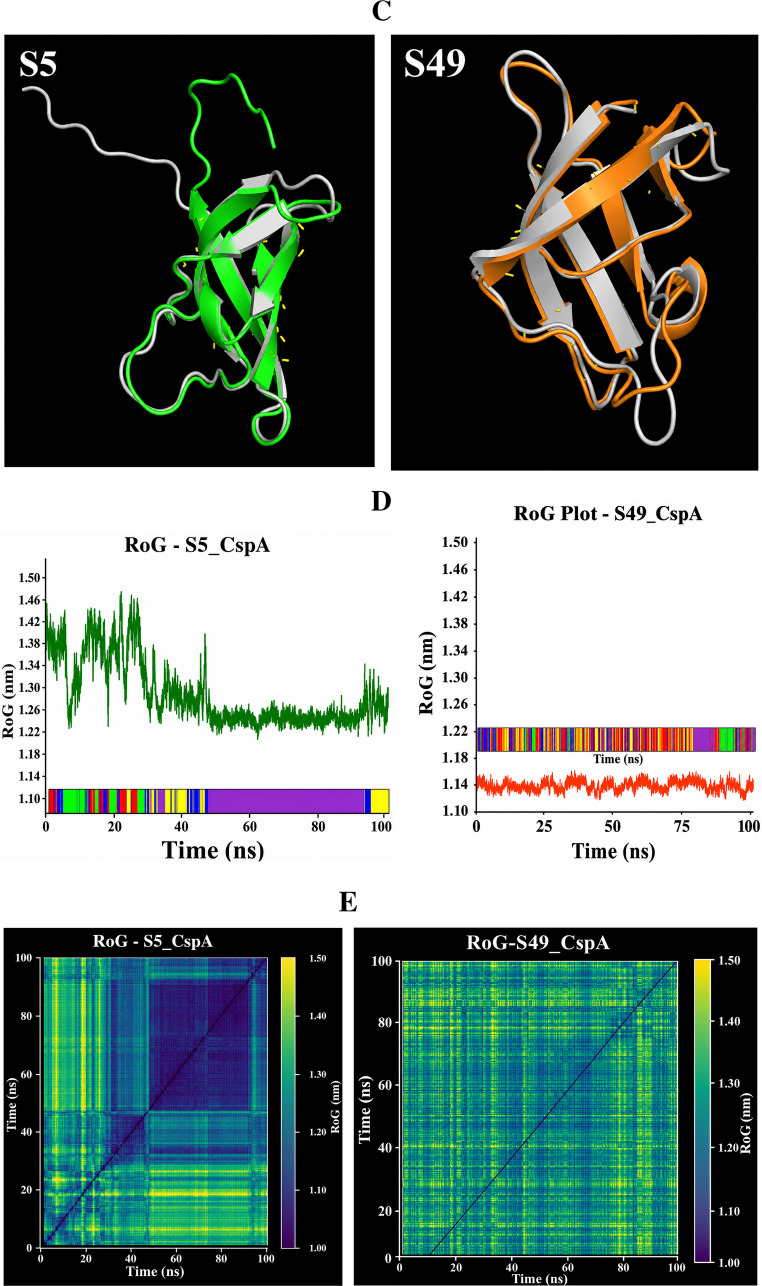
Molecular dynamics analysis of S5_CspA vs S49_CspA. **A** Root Mean Square Deviation (RMSD) of the backbone atoms across the simulation, measured relative to the initial structure. **B** Root Mean Square Fluctuation (RMSF) of residues in both proteins, highlighting regions of flexibility. **C** Structural alignment of the representative structure of each protein with the initial conformation to assess overall conformational changes. **D** Radius of Gyration (RoG) of both proteins throughout the simulation, with a linear cluster projection of the trajectory performed using TTClust. Each bar represents a frame, and the color indicates the cluster number. **E** Distance matrix plot visualizing pairwise structural deviations between frames, providing insights into conformational transitions. Figures of both proteins were generated using 50 ns due to protein size and memory limit.

Molecular dynamics analysis revealed notable differences in protein flexibility among RNA-binding proteins (RBPs). The RMSD plot for S5_CspA showed high fluctuation compared to S49_CspA, which was explained by the RMSF plot indicating substantial flexibility in the N-terminal region (Fig. 6). This suggests that S5_CspA may have a more adaptable binding surface, potentially allowing recognition of diverse RNA structures, whereas the more stable N-terminal of S49_CspA could favor interactions with structured or specific RNA sequences. Drug-resistant strains exhibited greater fluctuations in their RBPs, implying possible evolutionary adaptations that enhance RNA interaction and contribute to pathogenicity. Similar fluctuation patterns were observed in the core regions, highlighting the need to identify functional domains to interpret the effects of stability versus flexibility. Comparable dynamics were observed in CspB and S1_RBP proteins (Supplementary Materials S13 and S14).

## Discussion

Our genome-based analysis of 45 predicted multi-drug-resistant (MDR) and six predicted drug-sensitive *S. aureus* strains suggests that RNA-binding proteins (RBPs) may play critical roles in virulence, antibiotic resistance, and regulatory mechanisms. Strain classification was based solely on genomic predictions of resistance genes, and all functional inferences are limited to in silico analyses; gene presence may not necessarily reflect expression or phenotypic activity. Key virulence genes, including *hlgA*, *hlgB*, and *hlgC*, were predicted in all strains, encoding gamma-hemolysins that contribute to immune evasion and host cell lysis (D’Onofrio et al. 2023). The *edinB* gene was predicted in strain S13, suggesting a potential role in disrupting epidermal cell differentiation and facilitating skin infections. Variation in the number of predicted virulence genes may reflect differences in virulence potential related to clinical origin or antibiotic resistance profiles.

Prediction of resistance genes revealed a high prevalence of *mecA* in 44 of 51 strains, indicating widespread predicted methicillin resistance (MRSA) consistent with global trends (Batool et al. 2021). The *blaZ* gene, responsible for β-lactamase-mediated penicillin resistance, was predicted in 17 strains. Six strains (S46–S51) lacked both *mecA* and *blaZ*, classifying them as predicted drug-sensitive. The *fusc* gene, conferring fusidic acid resistance, was predicted in only one strain (S45), highlighting potential strain-specific resistance patterns and emphasizing the importance of regional genomic surveillance to capture diverse resistance profiles.

In agreement with Afzal et al. (2022), ST8 was among the most common sequence types in our study, particularly associated with hospital-acquired MRSA strains, highlighting its global relevance. The predominance of clonal complexes CC5 and CC8 among USA strains further emphasized regional dominance. The absence of assigned clonal complexes in some strains suggests novel or uncharacterized lineages, reflecting genetic variability within *S. aureus* populations. Phylogenetic clustering based on sequence type or clonal complex aligned with Német et al. (2020), indicating that sequence type is a stronger predictor of genetic relatedness than clinical or geographic factors.

Proteome-wide screening revealed that *S. aureus* encodes 166 distinct Pfam RNA-binding domains (RBDs), with the ATP-binding cassette (ABC) transporter domain the most abundant RNA-binding domain (RBD), reflecting its established role in nutrient transport, detoxification, and antibiotic resistance via ATP-driven substrate translocation. This enrichment in predicted MDR strains may be associated with antibiotic efflux mechanisms, consistent with previous reports (Feng et al. 2020; Yamazaki and Hirose 2015; Jones and George 2004; Higgins 2001). MDR strains generally exhibited larger proteomes, suggesting that increased protein diversity may support survival under antibiotic stress, while strain-specific differences, such as in S37, may reflect the acquisition or upregulation of resistance genes (Peng et al. 2019; Sulaiman et al. 2022).

ANCOVA analysis revealed that 82 RNA-binding domains may significantly influence *S. aureus* virulence, supporting Henderson and Payne (1994), who highlighted the role of transporters in bacterial pathogenicity. Domains associated with ribosomal and RNA metabolism functions, including Macrolide resistance or efflux proteins (Macro), Aminoacyl-tRNA synthetase class II (tRNA_synt_2), Ribosomal.L25p, and Pseudouridine synthase class 2 (PseudoU_synth), displayed larger effect sizes, suggesting their potential contribution to virulence regulation and infection dynamics.

Our findings also suggested that RNA-binding protein domains (RBPDs) may have a relation to *S. aureus* antibiotic resistance, particularly against aminoglycosides. Positive correlations were observed between key RBPDs, including ribosomal proteins, RNA polymerase subunits, and six tested RBPs (S1, CspA, CspB, RsmH, RsmI, and SpoVG), and aminoglycoside resistance, indicating they may contribute to resistance mechanisms (Jain 2008; Luong et al. 2022). In contrast, PBP-dimer (Penicillin-Binding Protein Dimer) and RVT1 (Reverse Transcriptase Domain 1) may have a relation to susceptibility, as they showed negative correlations with β-lactam and other antibiotic resistance, consistent with prior reports suggesting that RNA polymerase subunits can influence antibiotic sensitivity (Cramer 2019; Ishihama 1992).

Ribosomal proteins L17, L27A, and L30 showed substantial effect sizes in trimethoprim resistance, suggesting their potential involvement in resistance mechanisms, consistent with previous studies (Tenson 1997; Ero et al. 2021). Clustering of ribosomal proteins indicated shared pathways influencing resistance, aligning with reports that ribosomal alterations contribute to antibiotic resistance (Wilson et al. 2020). In fusidic acid resistance analysis, proteins such as IF3C displayed negative correlations, supporting potential common susceptibility mechanisms, possibly involving ribosome recycling or prevention of premature termination (Ero et al. 2021). Further studies are needed to clarify how these ribosomal proteins modulate fusidic acid susceptibility and their potential as therapeutic targets in combating *S. aureus* antibiotic resistance.

The identification of eight significant RBPs in *S. aureus* suggested they might play important roles in virulence and regulatory mechanisms, including biofilm formation and host invasion (Gualerzi et al. 2003; Duval et al. 2010; Eshwar et al. 2017). Predicted regulatory proteins such as Hfq and SpoVG may modulate gene expression via small RNA–mRNA interactions, contributing to virulence and antibiotic resistance (Nakao et al. 1995; Schumacher et al. 2002). and could represent potential therapeutic targets. Multiple sequence alignment indicated that RBPs including CvfB, Hfq, RsmH, RsmI, and SpoVG are highly conserved across strains, suggesting strong evolutionary constraints on their functional roles in *S.* *aureus* pathogenicity (Huang et al. 2003; Pei et al. 2019). Prediction also revealed that some strains carry multiple copies of the MetG gene, potentially enhancing antimicrobial resistance, while the absence of RsmI in strain S42 may reflect unique adaptive strategies. Notably, the invasive strain TW20 (S26, ST228), with predicted increased resistance, highlights the need to monitor specific lineages to guide targeted management strategies (Yi et al. 2018; Huang et al. 2003). These findings underscored the value of genomic prediction in understanding the evolutionary dynamics, virulence potential, and resistance mechanisms of *S. aureus*. The consistent presence and distribution of RNA-binding protein domains (RBPDs) across the eight identified RBPs in *S. aureus* align with previous studies highlighting the essential functions of conserved RBPDs in stress response and virulence, especially in the context of antibiotic resistance (Ghosh and Sowdhamini 2017).

Regarding dynamic simulations, increased N-terminal flexibility in S5_CspA may enhance RNA recognition by accommodating diverse structures, similar to mechanisms observed in polypyrimidine tract-binding protein 1 (PTBP1) (Pirakitikulr 2015; Holmqvist and Vogel 2018; Damberger et al. 2024). In contrast, the more compact S49_CspA may favor specific RNA interactions, resembling stable RBP structures in *Mycobacterium tuberculosis* (Arnvig and Young 2009). These structural dynamics may reflect evolutionary adaptations in drug-resistant strains, optimizing RBPs for dual roles in virulence regulation and antibiotic resistance, as seen in *Salmonella* spp. (Westermann 2018). N-terminal flexibility may also contribute to allosteric regulation, modulating RNA-binding affinity and specificity (Romanelli et al. 2013; Pina et al. 2022).

## Conclusion

This study suggests that RNA-binding proteins (RBPs) may be associated with the adaptability and pathogenicity of *S. aureus*, particularly in multi-drug-resistant (MDR) strains. Genome- and proteome-based analyses of 51 strains revealed correlations between predicted RBPs and the presence of virulence genes, resistance profiles, and SNP-based clonal complex groupings. Molecular dynamics simulations indicated that RBPs, including cold shock proteins and S1 RNA-binding domain proteins, may have structural variations, with MDR strains showing increased N-terminal flexibility that may be related to RNA recognition and stress adaptation.

Limitations include the computational nature of the study, limited dataset, and the absence of horizontal gene transfer analysis. In addition, further transcriptomic evaluations and functional validation are required to validate our results. Both ANOVA and ANCOVA were applied to assess differences and correlations in RNA-binding protein domain (RBPD) assuming linear relationships. It is worthwhile to try using machine learning ML models e.g. random forests in future studies which consider nonlinear correlations using a large dataset of *S. aureus* strains, potentially enhancing predictive power and identifying combinatorial effects not captured by linear models. Overall, these associations suggested that RBPs may serve as potential biomarkers or therapeutic targets, and experimental validation is required to confirm their functional relevance in *S. aureus* virulence and antibiotic resistance.

## Supplementary Information

Below is the link to the electronic supplementary material.

ESM 1 Supplementary Material 1 (XLSX 17.6 KB)

ESM 2 Supplementary Material 2 (XLSX 105 KB)

ESM 3 Supplementary Material 3 (DOCX 9.71 MB)

## Data Availability

All pertinent data are provided within the manuscript and the supplementary file.
