# Supplementary material for: Comparative genomic analysis of RNA-binding proteins across some drug resistant and sensitive Staphylococcus aureus
Source: Appl Microbiol Biotechnol. 2026 Jun 24;110(1):189. doi: 10.1007/s00253-026-13906-x (PMC13294327; doi:10.1007/s00253-026-13906-x)
Supplement: Supplementary file 3 — Supplementary Material 3 (DOCX 9.71 MB) [file 253_2026_13906_MOESM3_ESM.docx]

**Supplementary materials**

**Supplementary Material S3.** Number of virulence genes per strain.


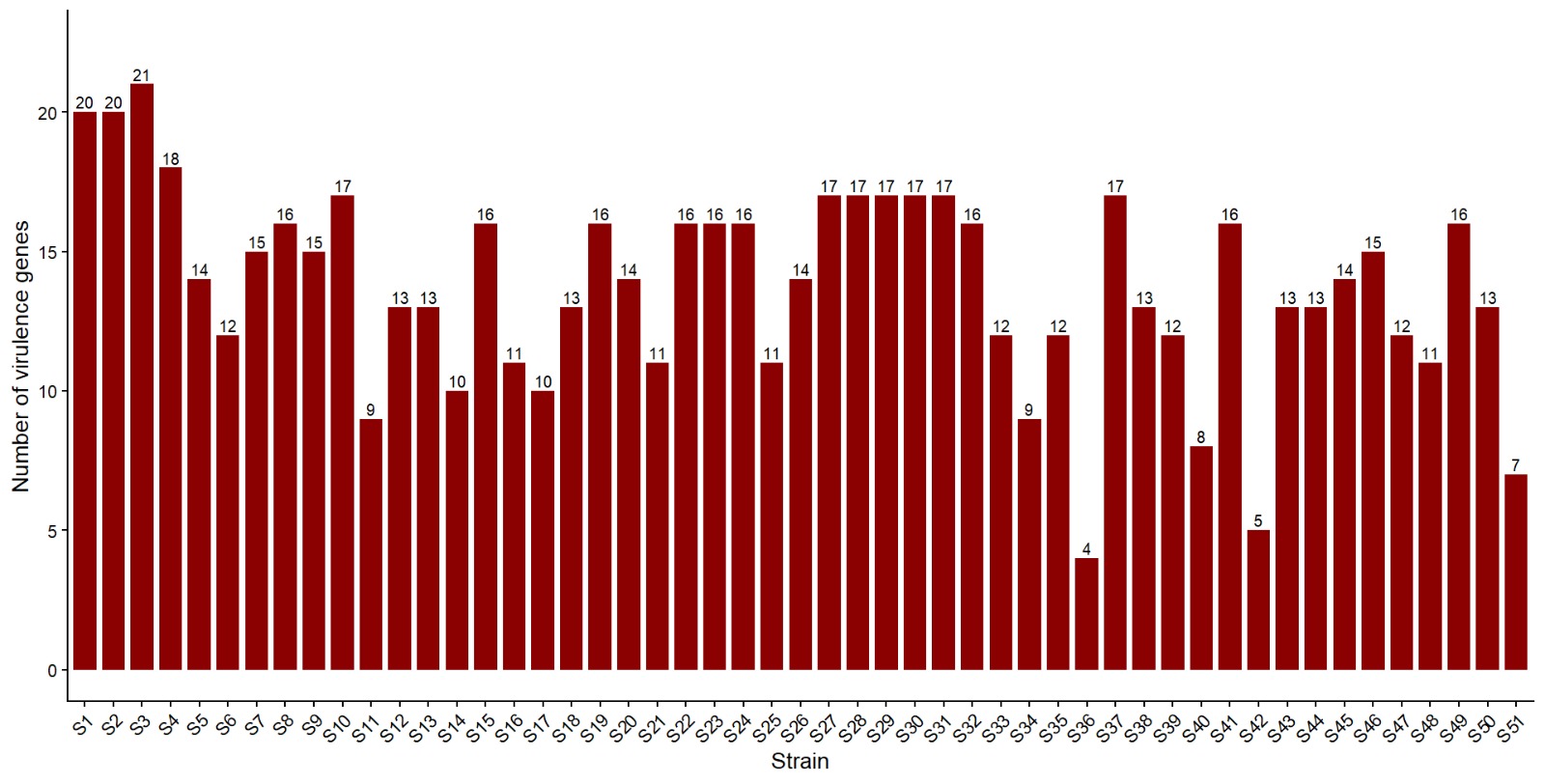


**Supplementary Material S4. (A)** Antimicrobials to which *S. aureus* strains were resistant; **(B)** The number of multi-drug resistance genes per strain.

**A**

**B**


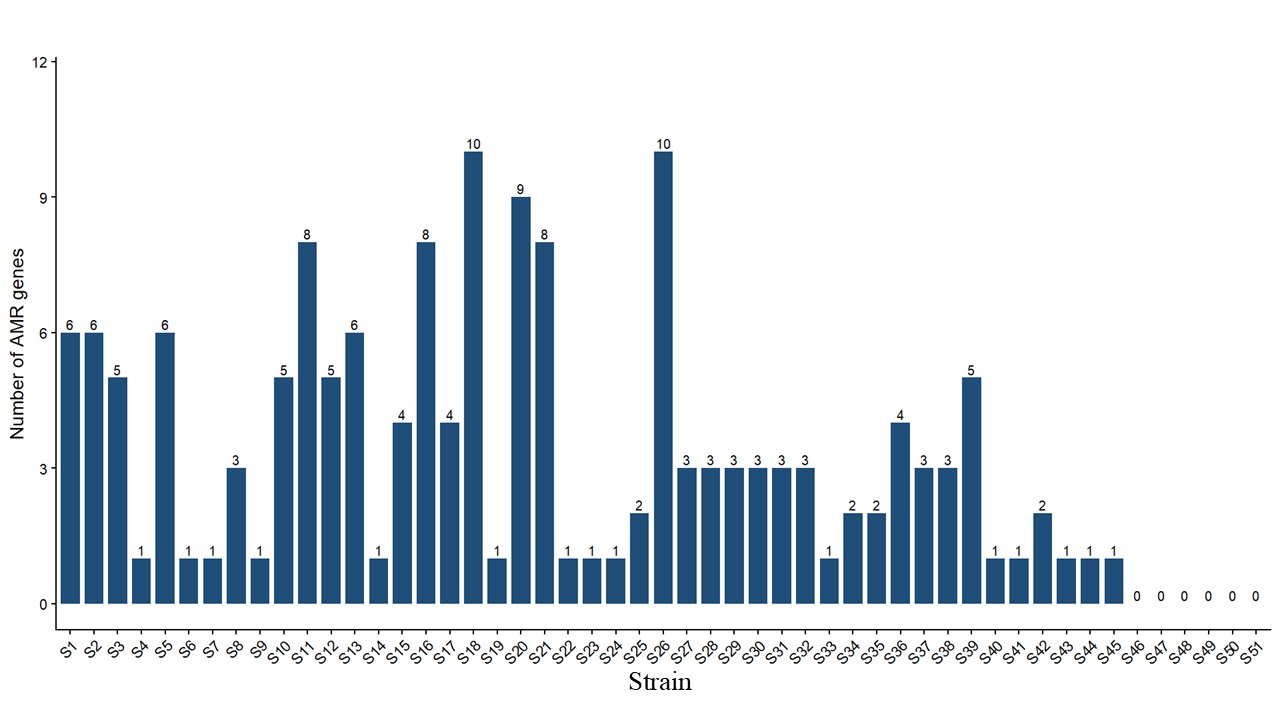


**Supplementary Material S5.** The ratio of RNA-binding protein domains to the total protein domains in each strain.

**
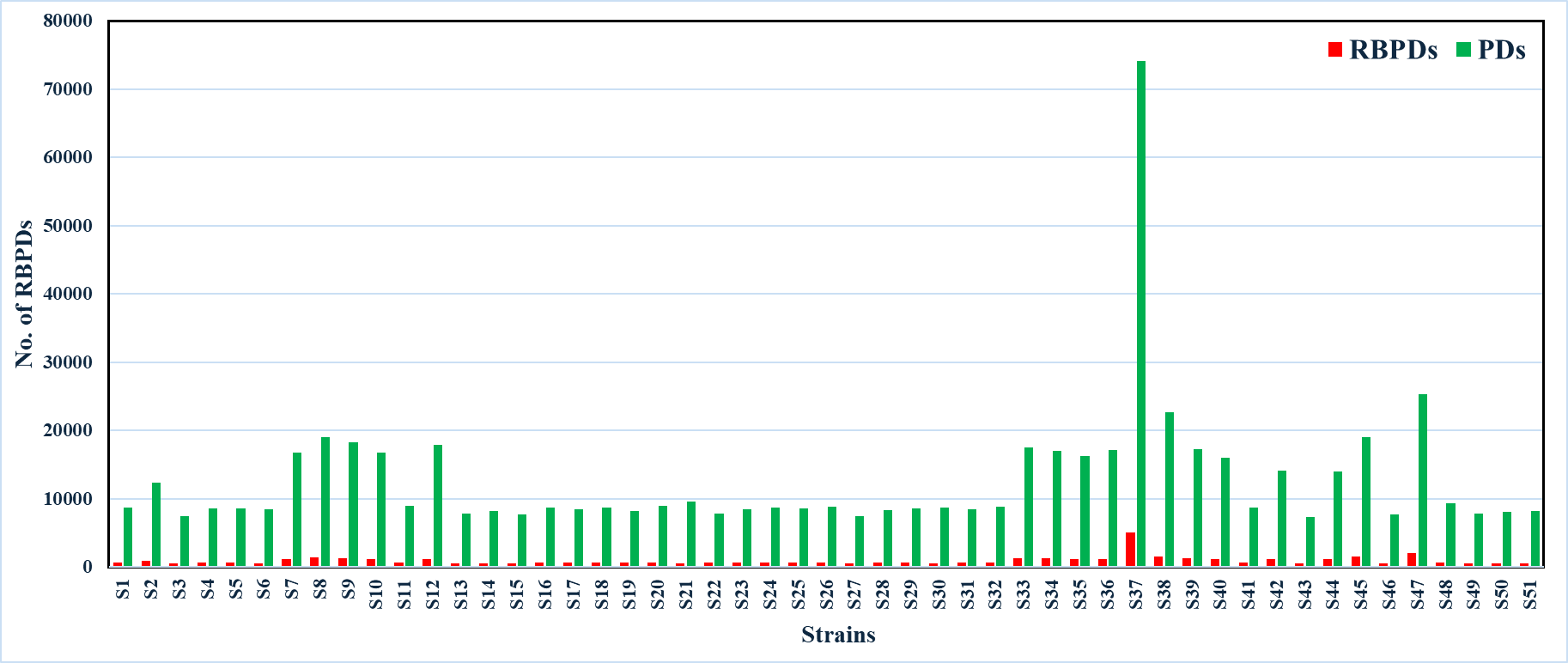
**

**Supplementary Material S6**. Ribosomal and Metabolic RNA-Binding Protein Domains (RBPDs) Positively Correlated with *S. aureus* Virulence

| Type of RBPDs | RBPDs | Function |
| --- | --- | --- |
| Ribosomal RBPDs | Ribosomal_L25p | These domains are involved in ribosome assembly, translation, and protein synthesis, playing a crucial role in bacterial growth and adaptation to host environments. |
|  | Ribosomal_S7 |  |
|  | Ribosomal_L16 |  |
|  | Ribosomal_S2 |  |
|  | Ribosomal_S4 |  |
|  | Ribosomal_L3 |  |
|  | Ribosomal_L2 |  |
|  | Ribosomal_L4 |  |
|  | Ribosomal_L5 |  |
|  | Ribosomal_L10 |  |
| Metabolic RBPDs | Macro | These domains participate in essential cellular processes, such as antibiotic resistance (Macro), tRNA synthesis (tRNA_synt_2), RNA modification (PseudoU_synth_2, SpoU_methylase), RNA degradation (RNase_T), and energy metabolism (GTP_EFTU, Aconitase), which can contribute to bacterial virulence and survival. |
|  | tRNA_synt_2 |  |
|  | PseudoU_synth_2 |  |
|  | RNase_T |  |
|  | SpoU_methylase |  |
|  | GTP_EFTU |  |
|  | Aconitase |  |

**Supplementary Material S7.** RBPDs correlated with aminoglycoside resistance.

| **Category** | **RBPDs** | **Function** |
| --- | --- | --- |
| **Ribosomal Proteins** | Ribosomal.S11, Ribosomal.L10, Ribosomal.L6, Ribosomal.S5, Ribosomal.L2 | Core components of ribosomes were involved in protein synthesis. |
| **RNA-Processing Proteins** | Ribonuclease_P, TruB.N, RNA.pol.Rpb2 | Processing and modification of RNA molecules, including tRNA and mRNA. |
| **Translation Factors** | EF_TS, GTP_EFTU | Facilitate various stages of protein synthesis (e.g., elongation, initiation). |
| **RNA-interacting Proteins** | HfQ, tRNA.bind, S15_NS1_EPRS_RNA.bind | Bind to RNA for stability, processing, or regulatory functions. |
| **Helicases** | DEAD, UvrD.helicase, DEAD.like_helicase_N | Unwind DNA/RNA helices during replication, repair, and transcription. |
| **Pseudouridine Synthases** | PseudoU.synth.2, SpoU.methylase | Modify RNA by synthesizing pseudouridine for structural stability. |
| **Protein Modification** | PCMT, Peptidase.S7, TP_methylase | Modify and stabilize proteins post-translationally. |
| **Nucleotide Metabolism** | ADK, Ribonuclease_H, Rrf | Synthesize or process nucleotides essential for DNA/RNA metabolism. |
| **DNA/RNA Polymerases** | RNA.pol.Rpb1_3, RNA.pol.A.Ctd | Catalyze the synthesis of RNA and DNA during replication and transcription. |
| **Metabolic & Transport** | CorA, ATP.synt.ab, SecY | Facilitate metabolism and transport within the cell. |

**Supplementary Material S8.** RNA-binding proteins (RBPs) identified in *S. aureus*, describing their functions, roles in bacteria, and relevant references.

| **RBPs** | **Description** | **Role in *S. aureus*** | **References** |
| --- | --- | --- | --- |
| **CspA** | Cold shock protein A | Affects biofilm development, protease production, and cold shock response | **(Gualerzi *et al.* 2003); (Duval *et al.* 2010)** |
| **CspB** | Cold shock protein B | Impacts growth, multiplication in the host environment, and host cell invasion | **(Gualerzi *et al.* 2003)**; **(Duval *et al.* 2010)**; **(Eshwar *et al.* 2017)** |
| **CvfB** | Conserved Virulence Factor B | Regulates the expression of virulence genes through RNA binding | **(Bycroft *et al.* 1997, Liang *et al.* 2017)** |
| **Hfq** | Regulatory protein, Sm-like family | Facilitates pairing of sRNAs with target mRNAs, modulates mRNA degradation, possibly RNA transcription; implicated in virulence in other pathogens | **(Schumacher *et al.* 2002))(Nakao *et al.* 1995)** |
| **RsmI and RsmH** | Ribosomal methyltransferases | Contribute to oxidative stress resistance and translation fidelity under stress conditions | **(Kaito *et al.* 2005, Kimura and Suzuki 2010)** |
| **CvfD (S1 RNA-binding Protein)** | RNA-binding protein regulator | Controls expression of PhoU2 master regulator, involved in cold sensitivity and mRNA/sRNA level regulation; facilitates ribosomal interactions with mRNA | **(Hajnsdorf and Boni 2012); (Holmqvist and Vogel 2018)** |
| **SpoVG** | Stage V Sporulation Protein G | Influences production of capsule, extracellular nuclease, protease, and lipase; involved in methicillin and glycopeptide resistance | **(Frisby and Zuber 1991); (Schulthess *et al.* 2009); (Pan *et al.* 2014); (Liu *et al.* 2016)** |

**Supplementary Material S9.** Multiple sequence alignment of the eight RNA binding proteins in *S. aureus* strains. **(A)** CspA; **(B)** CspB; **(C)** CvfB; **(D)** HfQ; **(E)** RsmH; **(F)** RsmI; **(G)** S1_RBP; **(H)** SpoVG.

**A**

**
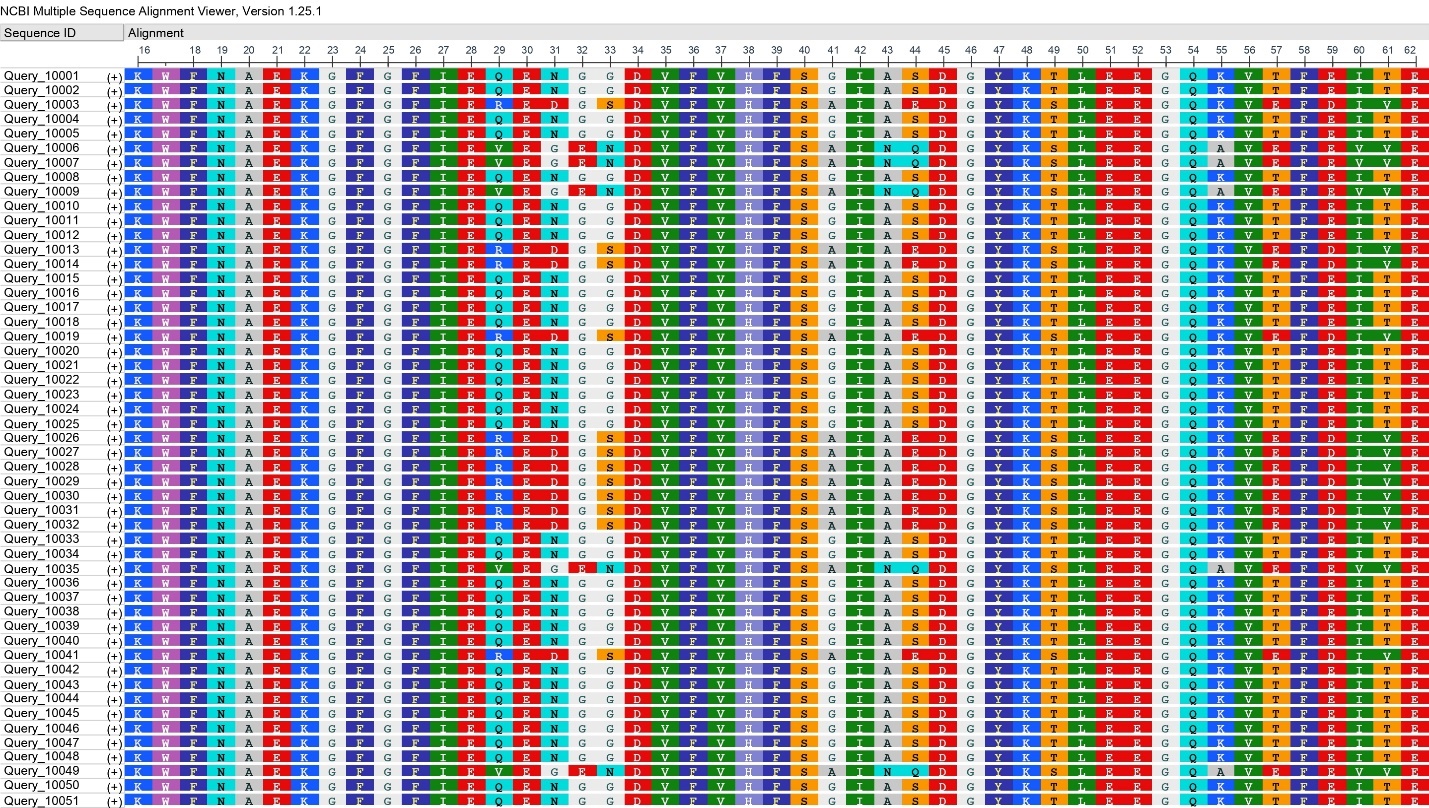
**

**B**

**
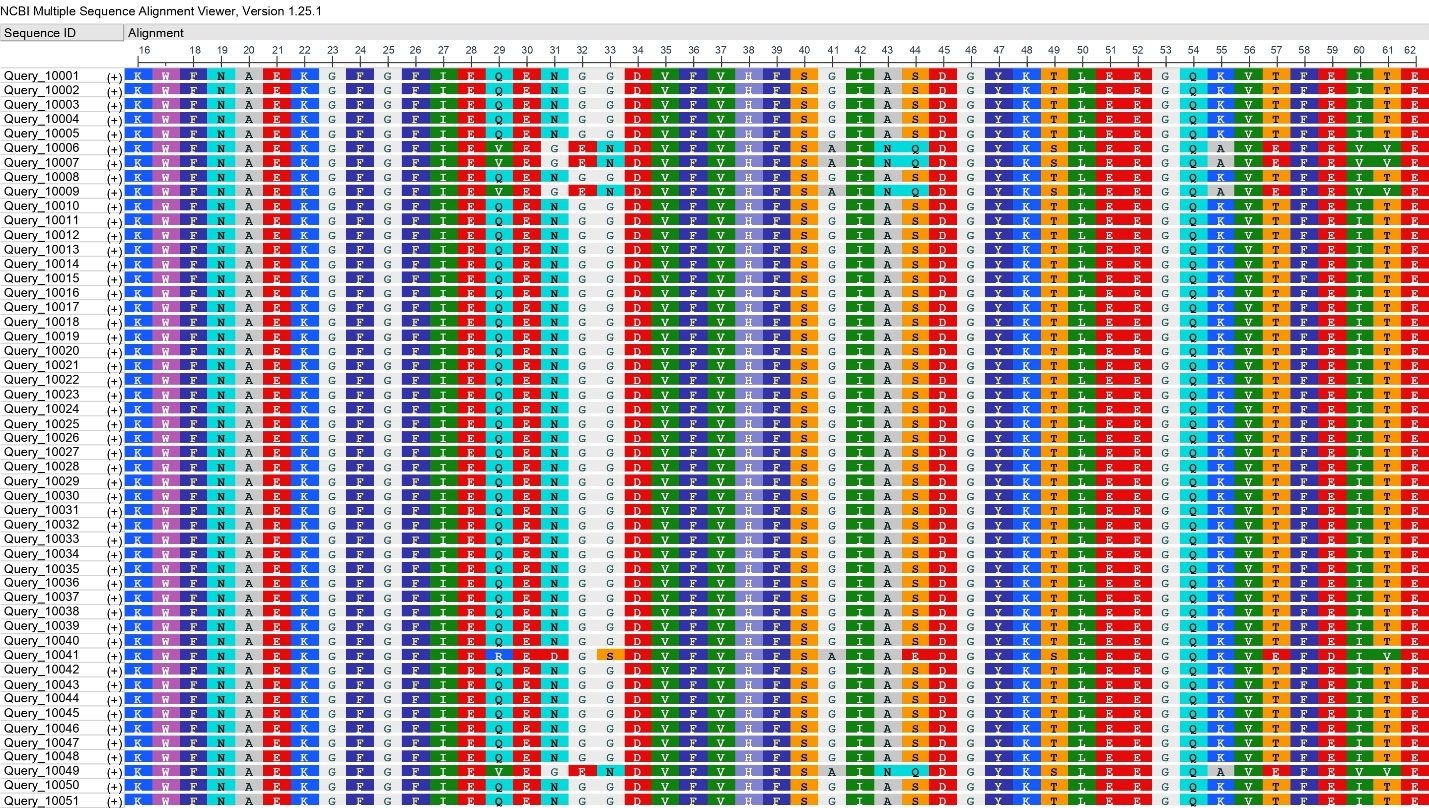
**

**C
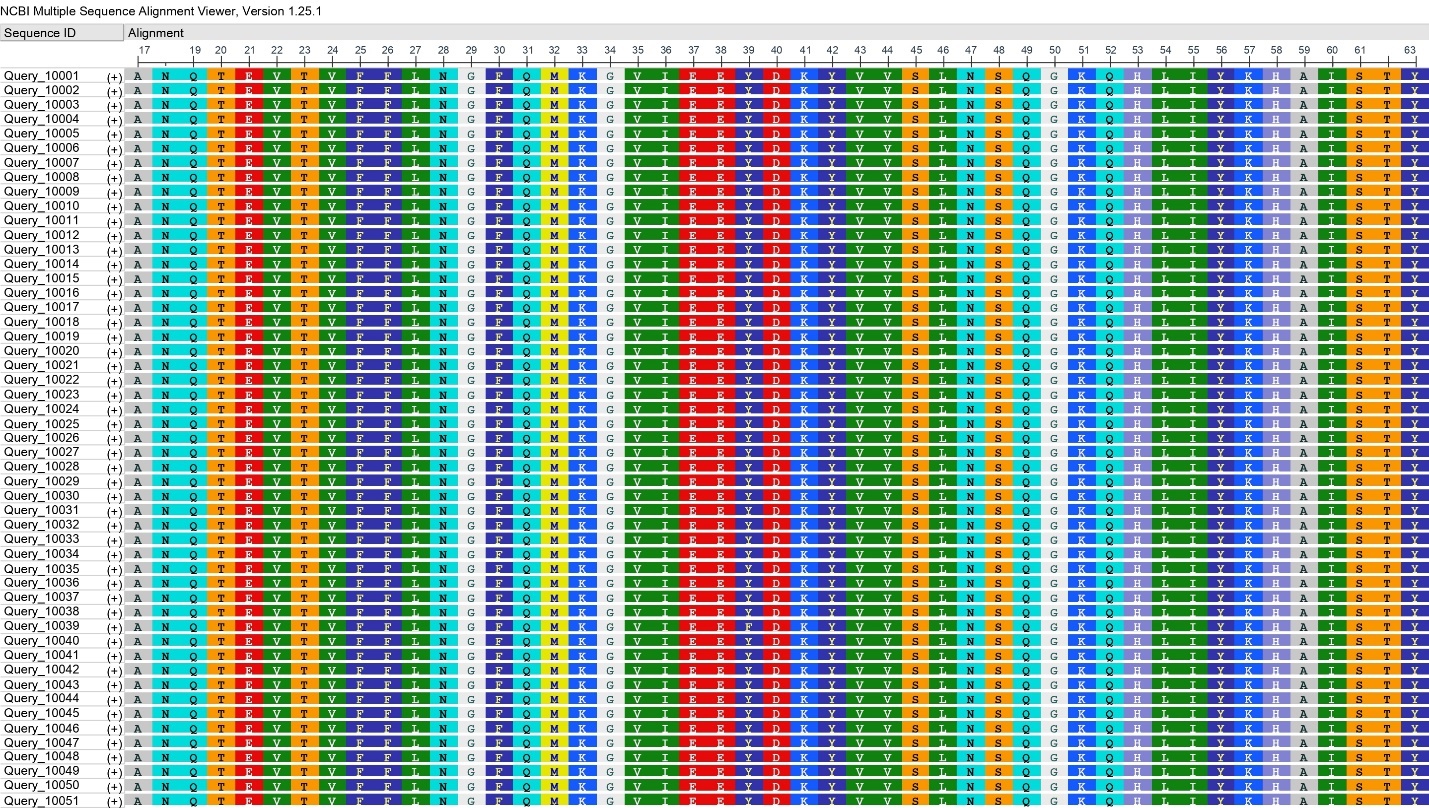
**

**D**

**
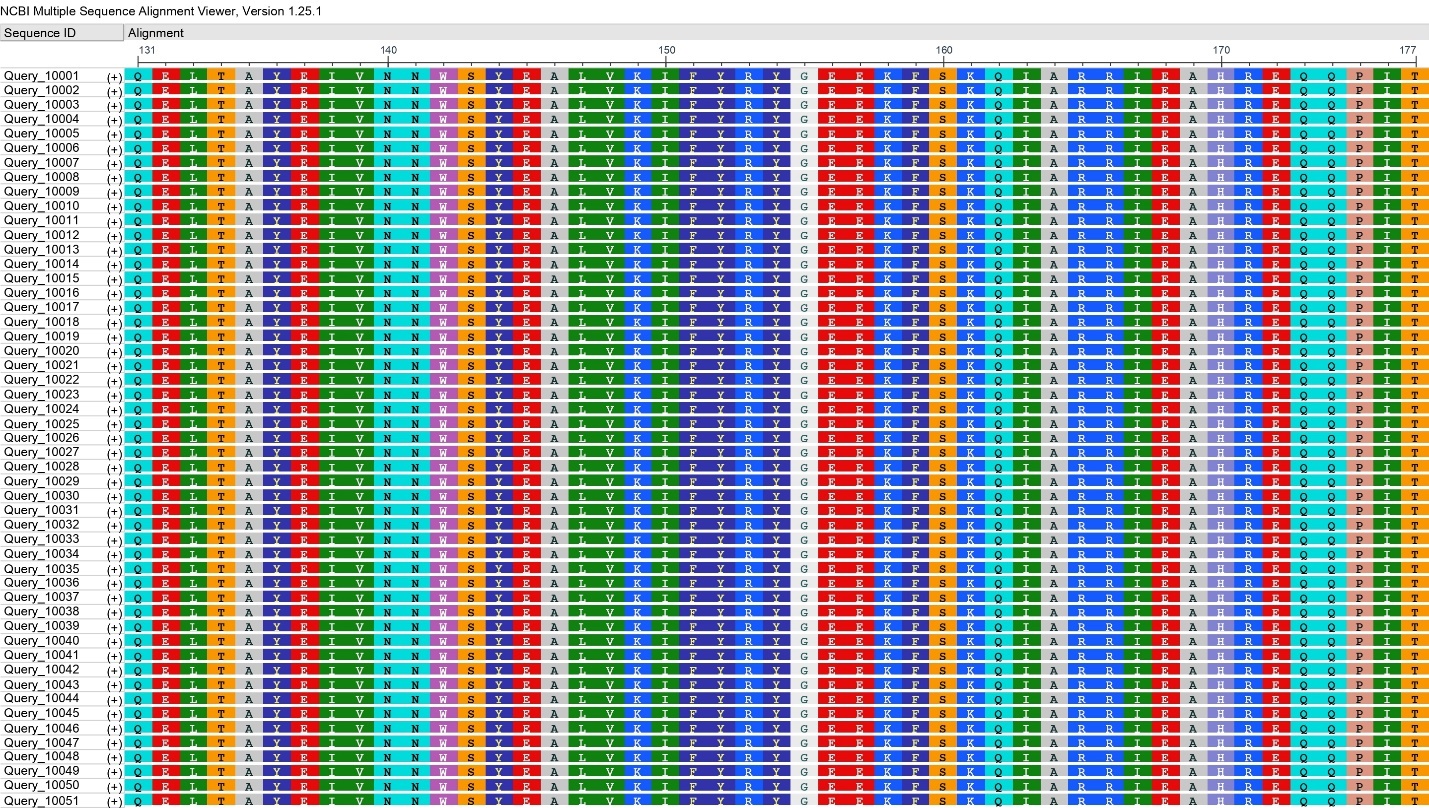
**

**E**

**
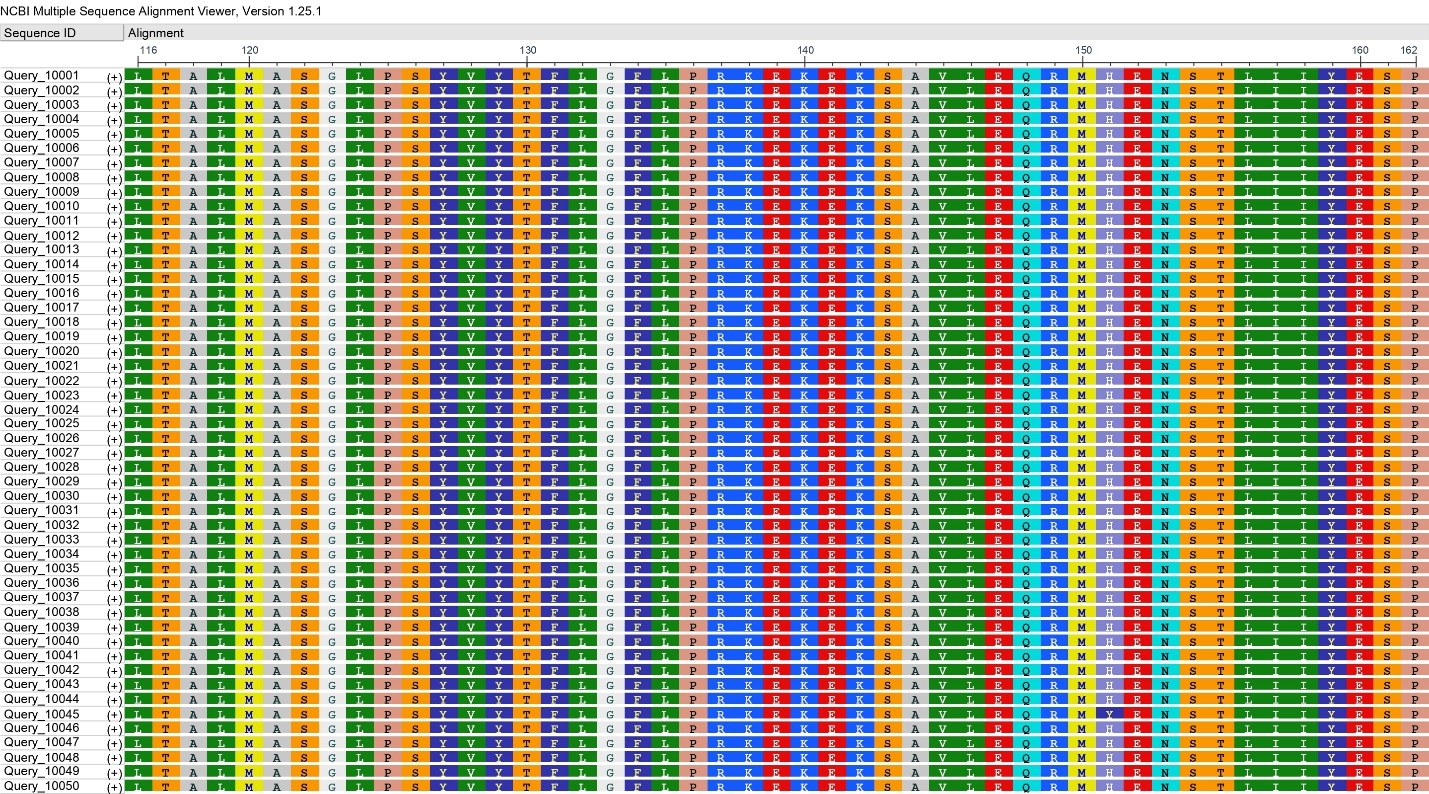
**

**F**

**
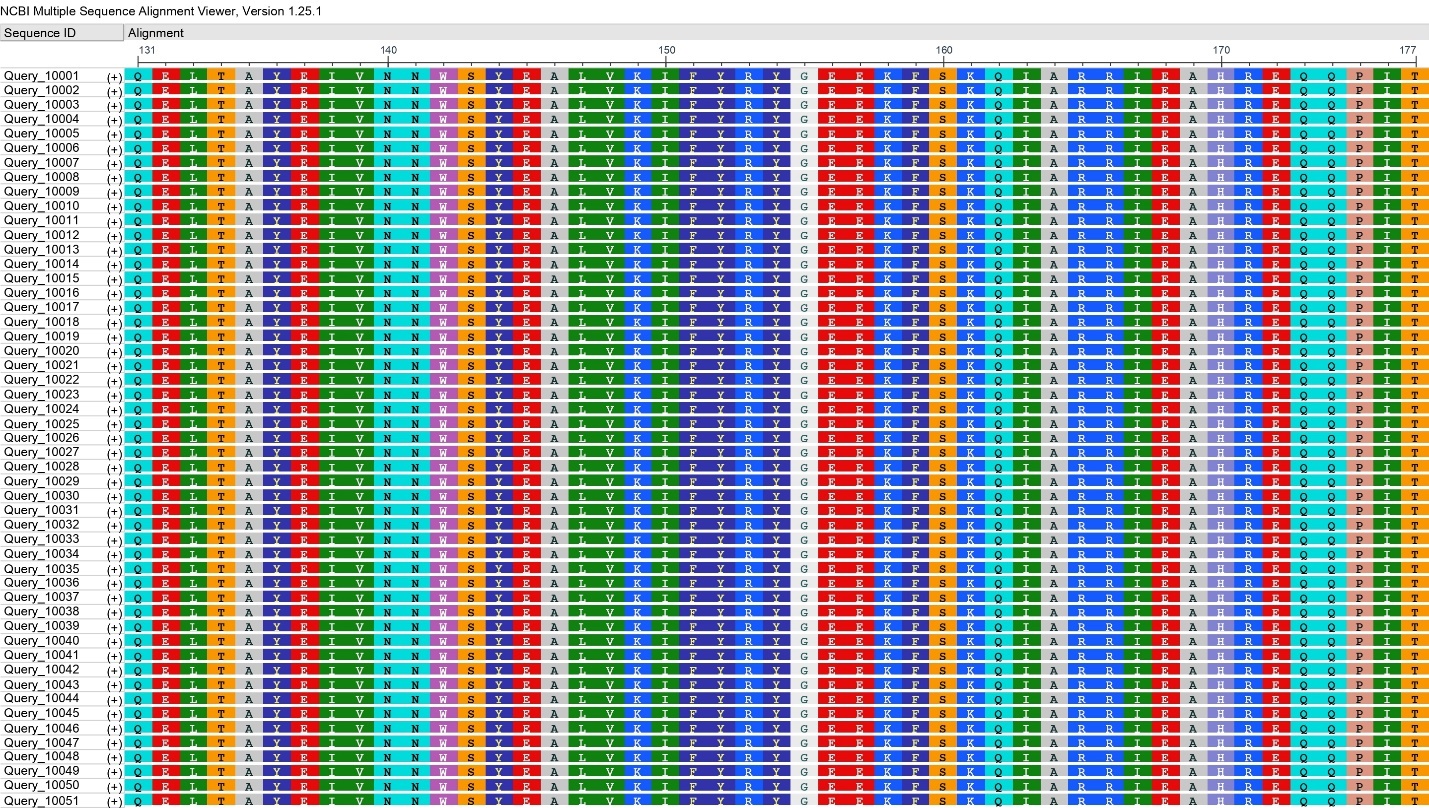
**

**G**

**
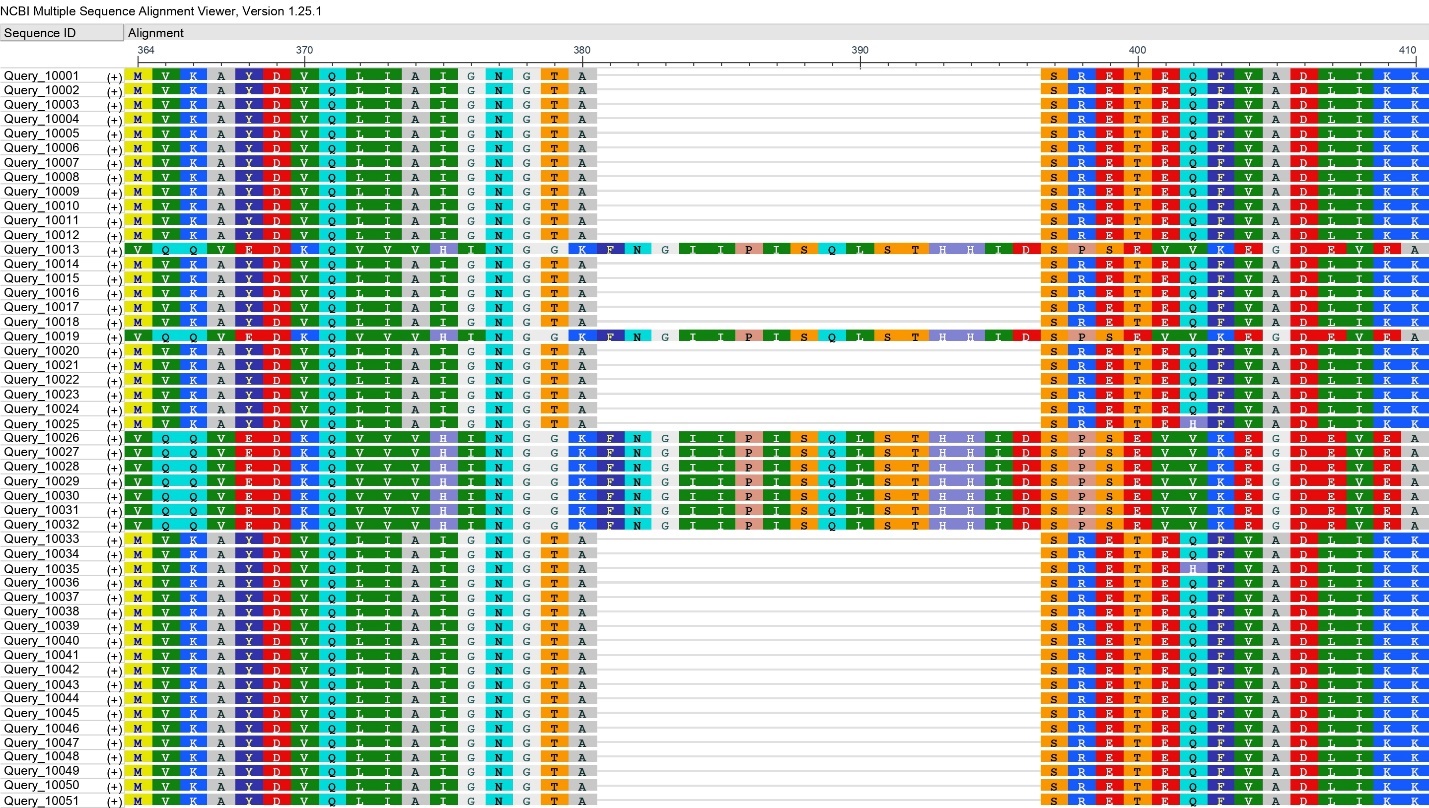
**

**H**

**
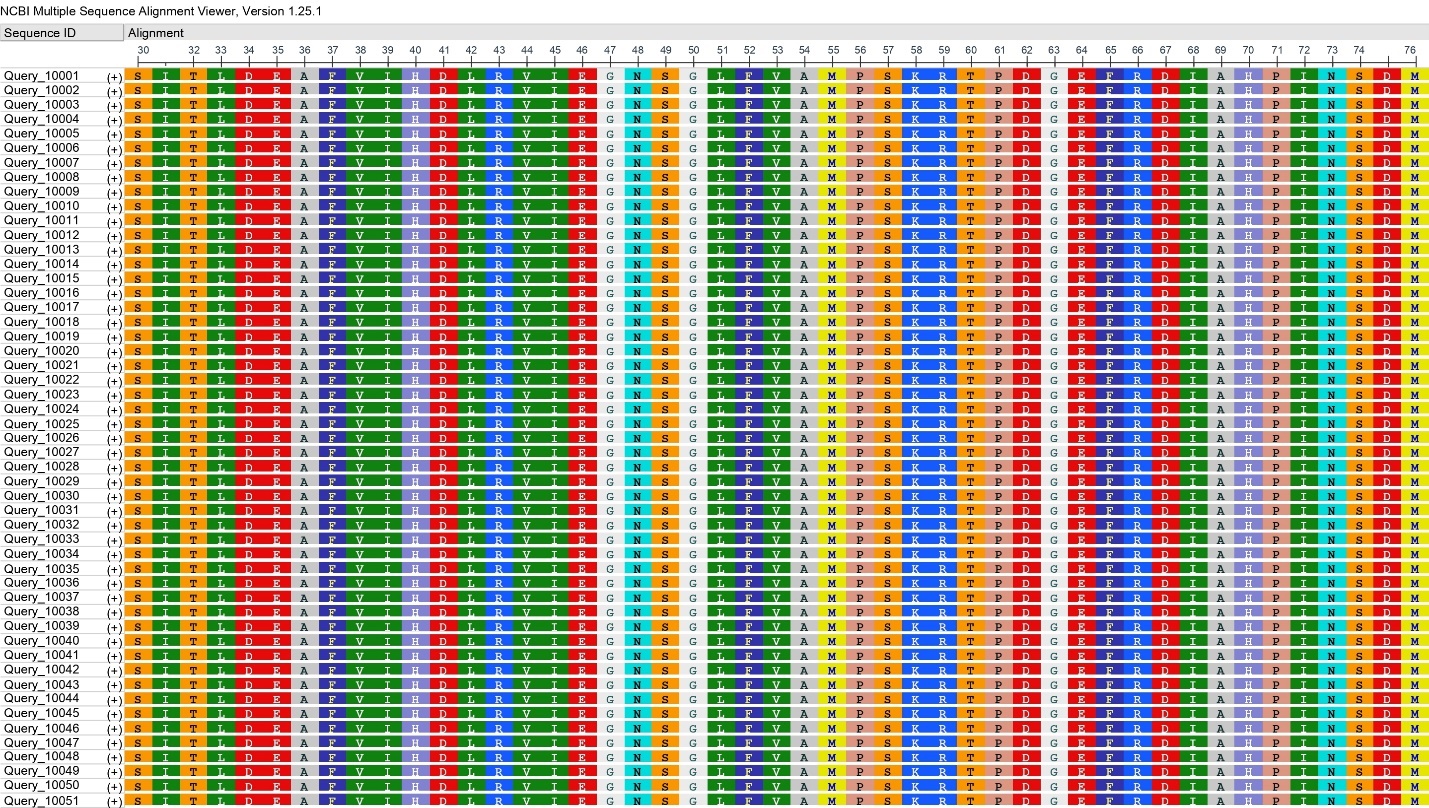
**

**Supplementary Material S10.** Multiple sequence alignment of CspA, CspB, and S1_RBP across *S. aureus* strains.

| **RBP** | **Conserved Regions** | **Strains with Differences** | **Unique region** |
| --- | --- | --- | --- |
| CspA | 1^st^ to 28^th^ amino acid region and from 46^th^ to 57^th^ amino acid region | S3, S6, S7, S9, S19, S26, S27, S28, S29, S30, S31, S32, S35, S41, S48 | Region from 29^th^ to 45^th^ amino acids |
| CspB | 1^st^ to 28^th^ amino acid region, from 34^th^ to 42^nd^ amino acid region, and from 45^th^ to 54^th^ amino acid region | S6, S7, S9, S41, S 49 | Regions from 29^th^ to 33^th^ amino acids, between 43^th^ and 44^th^ amino acids |
| S1_RBP | From S1 to S12, S20 to S25 and S33 to S51 | S13, S19, S26, S27, S28, S29, S30, S31, S32 | Entire sequence |

**Supplementary Material S11.** Distribution of the RBPDs of the eight RBPs identified in the tested *S. aureus* strains.

| **Strains** | **CSD** | **CvfB** | **HfQ** | **Methyl transf** | **AdoMet MTases** | **TP methylase** | **S1** | **Tex** | **HHH** | **YqgF** | **RuvC** | **T2SSK** | **SpoVG** |
| --- | --- | --- | --- | --- | --- | --- | --- | --- | --- | --- | --- | --- | --- |
| **S1** | 3 | 1 | 1 | 14 | 26 | 6 | 1 | 15 | 9 | 1 | 2 | 3 | 1 |
| **S2** | 5 | 1 | 1 | 21 | 40 | 6 | 1 | 15 | 14 | 1 | 3 | 3 | 1 |
| **S3** | 3 | 1 | 1 | 11 | 30 | 6 | 1 | 15 | 9 | 1 | 2 | 3 | 1 |
| **S4** | 3 | 1 | 1 | 12 | 22 | 6 | 1 | 19 | 9 | 0 | 1 | 3 | 1 |
| **S5** | 3 | 1 | 1 | 14 | 26 | 6 | 1 | 19 | 10 | 1 | 2 | 3 | 1 |
| **S6** | 3 | 1 | 1 | 13 | 19 | 6 | 1 | 19 | 9 | 1 | 2 | 3 | 1 |
| **S7** | 6 | 1 | 1 | 23 | 43 | 12 | 1 | 38 | 18 | 1 | 3 | 6 | 1 |
| **S8** | 6 | 1 | 1 | 21 | 55 | 12 | 1 | 42 | 18 | 2 | 4 | 6 | 1 |
| **S9** | 5 | 1 | 1 | 21 | 50 | 16 | 1 | 40 | 17 | 1 | 2 | 6 | 1 |
| **S10** | 6 | 1 | 1 | 22 | 48 | 12 | 1 | 34 | 18 | 1 | 4 | 6 | 1 |
| **S11** | 3 | 1 | 1 | 14 | 27 | 6 | 1 | 19 | 9 | 1 | 2 | 3 | 1 |
| **S12** | 6 | 1 | 1 | 26 | 47 | 12 | 1 | 38 | 18 | 2 | 4 | 6 | 1 |
| **S13** | 3 | 1 | 1 | 11 | 19 | 6 | 1 | 19 | 9 | 1 | 2 | 3 | 1 |
| **S14** | 3 | 1 | 1 | 14 | 21 | 6 | 1 | 17 | 10 | 1 | 2 | 3 | 1 |
| **S15** | 3 | 1 | 1 | 11 | 20 | 6 | 1 | 4 | 10 | 1 | 2 | 3 | 1 |
| **S16** | 3 | 1 | 1 | 13 | 25 | 6 | 1 | 19 | 9 | 1 | 2 | 3 | 1 |
| **S17** | 3 | 1 | 1 | 15 | 23 | 6 | 1 | 17 | 10 | 1 | 2 | 3 | 1 |
| **S18** | 3 | 1 | 1 | 11 | 28 | 6 | 1 | 19 | 9 | 1 | 1 | 3 | 1 |
| **S19** | 3 | 1 | 1 | 10 | 20 | 6 | 1 | 19 | 9 | 1 | 2 | 3 | 1 |
| **S20** | 3 | 1 | 1 | 13 | 26 | 6 | 1 | 17 | 9 | 1 | 2 | 2 | 1 |
| **S21** | 3 | 1 | 1 | 15 | 24 | 6 | 1 | 19 | 9 | 1 | 2 | 3 | 1 |
| **S22** | 3 | 1 | 1 | 12 | 22 | 6 | 1 | 19 | 9 | 1 | 2 | 3 | 1 |
| **S23** | 3 | 1 | 1 | 12 | 22 | 6 | 1 | 19 | 9 | 1 | 1 | 3 | 1 |
| **S24** | 3 | 1 | 1 | 11 | 22 | 6 | 1 | 19 | 9 | 1 | 2 | 3 | 1 |
| **S25** | 3 | 1 | 1 | 14 | 24 | 6 | 1 | 19 | 9 | 1 | 2 | 3 | 1 |
| **S26** | 3 | 1 | 1 | 14 | 26 | 6 | 1 | 19 | 9 | 0 | 1 | 3 | 1 |
| **S27** | 3 | 1 | 1 | 14 | 28 | 6 | 1 | 19 | 9 | 1 | 2 | 3 | 1 |
| **S28** | 3 | 1 | 1 | 13 | 28 | 6 | 1 | 19 | 9 | 1 | 1 | 3 | 1 |
| **S29** | 3 | 1 | 1 | 15 | 28 | 6 | 1 | 19 | 9 | 1 | 2 | 3 | 1 |
| **S30** | 3 | 1 | 1 | 15 | 29 | 6 | 1 | 19 | 9 | 0 | 2 | 3 | 1 |
| **S31** | 3 | 1 | 1 | 15 | 29 | 6 | 1 | 19 | 9 | 1 | 2 | 3 | 1 |
| **S32** | 3 | 1 | 1 | 12 | 25 | 6 | 1 | 19 | 9 | 1 | 2 | 3 | 1 |
| **S33** | 6 | 1 | 1 | 27 | 49 | 12 | 1 | 38 | 18 | 2 | 4 | 6 | 1 |
| **S34** | 6 | 1 | 1 | 26 | 46 | 12 | 1 | 38 | 18 | 2 | 4 | 6 | 1 |
| **S35** | 5 | 1 | 1 | 26 | 47 | 12 | 1 | 38 | 18 | 2 | 4 | 6 | 1 |
| **S36** | 6 | 1 | 1 | 20 | 47 | 12 | 1 | 38 | 18 | 1 | 2 | 6 | 1 |
| **S37** | 3 | 1 | 1 | 126 | 272 | 60 | 1 | 136 | 90 | 7 | 8 | 30 | 1 |
| **S38** | 6 | 1 | 1 | 29 | 62 | 18 | 1 | 49 | 28 | 1 | 3 | 9 | 1 |
| **S39** | 6 | 1 | 1 | 32 | 54 | 12 | 1 | 38 | 18 | 1 | 3 | 6 | 1 |
| **S40** | 6 | 1 | 1 | 25 | 41 | 12 | 1 | 34 | 20 | 2 | 3 | 6 | 1 |
| **S41** | 3 | 1 | 1 | 12 | 23 | 6 | 1 | 19 | 9 | 1 | 1 | 3 | 1 |
| **S42** | 6 | 1 | 1 | 16 | 44 | 12 | 1 | 38 | 18 | 1 | 3 | 6 | 1 |
| **S43** | 3 | 1 | 1 | 12 | 24 | 6 | 1 | 19 | 9 | 1 | 2 | 3 | 1 |
| **S44** | 6 | 1 | 1 | 16 | 40 | 12 | 1 | 38 | 20 | 2 | 4 | 6 | 1 |
| **S45** | 8 | 1 | 1 | 22 | 48 | 12 | 1 | 55 | 20 | 3 | 6 | 9 | 1 |
| **S46** | 3 | 1 | 1 | 13 | 23 | 6 | 1 | 19 | 9 | 1 | 2 | 3 | 1 |
| **S47** | 9 | 1 | 1 | 35 | 64 | 18 | 1 | 61 | 27 | 3 | 6 | 10 | 1 |
| **S48** | 3 | 1 | 1 | 13 | 22 | 6 | 1 | 19 | 9 | 1 | 0 | 3 | 1 |
| **S49** | 3 | 1 | 1 | 10 | 19 | 6 | 1 | 19 | 9 | 0 | 0 | 3 | 1 |
| **S50** | 5 | 1 | 1 | 9 | 22 | 6 | 1 | 19 | 9 | 1 | 0 | 3 | 1 |
| **S51** | 2 | 1 | 1 | 7 | 19 | 6 | 1 | 19 | 9 | 1 | 0 | 3 | 1 |

**CSD**: Cold Shock Domain; **CvfB**: Conserved Virulence Factor B; **HfQ**: Host Factor protein Q.; **Methyl transf**: Methyltransferase; **AdoMet MTases**: S-Adenosylmethionine-dependent Methyltransferases; **TP methylase**: Thiopurine Methyltransferase; **S1**: Ribosomal Protein S1 Domain; **Tex**: Transcription-Excision Factor; **HHH**: Helix-Hairpin-Helix; **YqgF**: A conserved DNA/RNA processing endonuclease; **RuvC**: Resolvase U Virulence C; **T2SSK**: Type II Secretion System Protein K; **SpoVG**: Stage V Sporulation Protein G.

**Supplementary Material S12.** The consistent distribution of RNA-binding protein domains (RBPDs) among the eight identified RBPs across *S. aureus* strains.

**
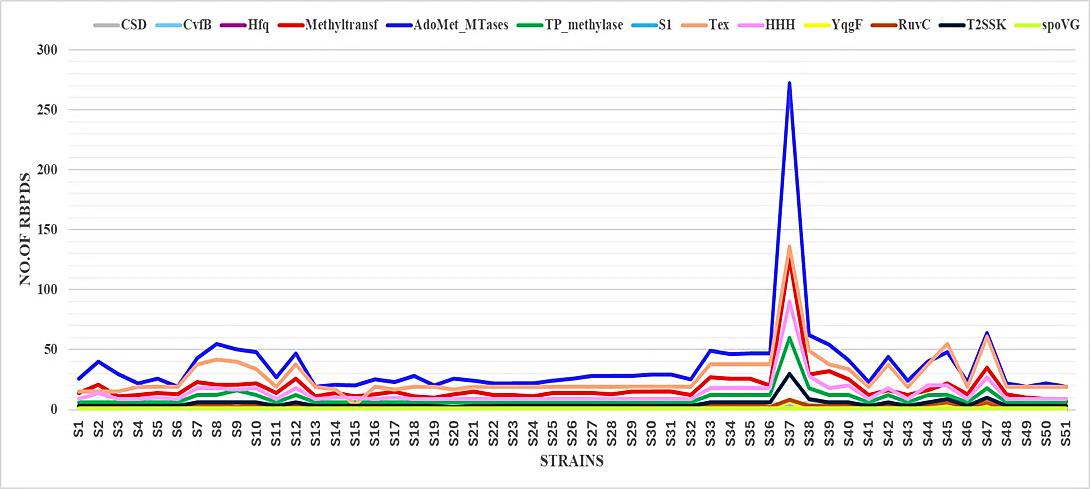
**

**Supplementary Material S13.** Molecular dynamics analysis of S15_CspB vs S49_CspB. (**A)** RMDS of the backbone of the trajectories relative to the backbone of the initial structure. (**B)** RMSF of the residues in both proteins. (**C)** The representative structure of each protein was structurally aligned with the initial structure. (**D)** RoG of both proteins across the simulation with a linear projection of cluster was performed for the trajectory using ttclust. Every bar line represented a frame, and the color was a cluster number. (**E)** Plot of the distance matrix allowed the distance between two frames to be visualized.


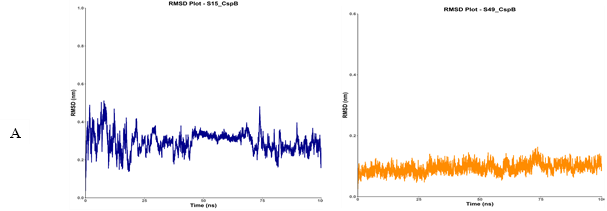


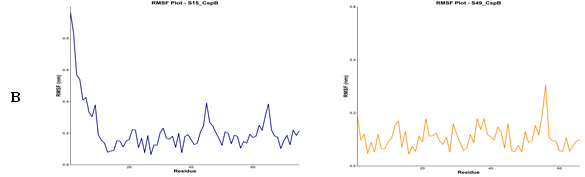

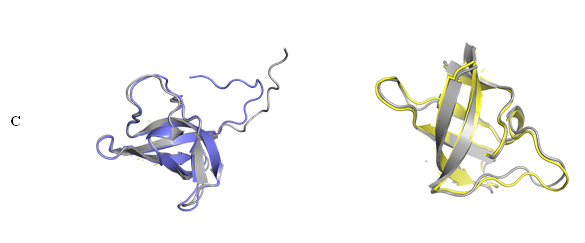


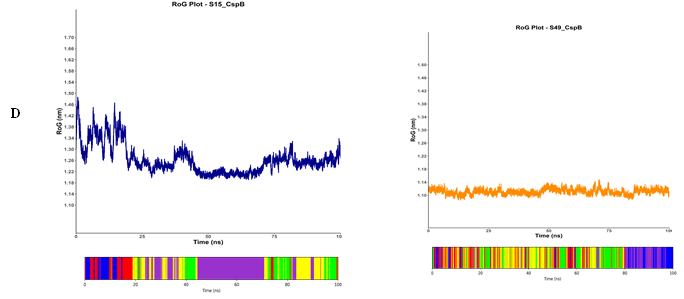


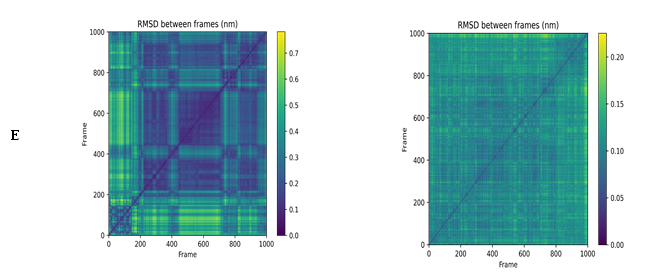


**Supplementary Material S14.** Molecular dynamics analysis of S26_S1 vs S51_S1. Figures of both proteins were generated using 50 ns due to protein size and memory limit.

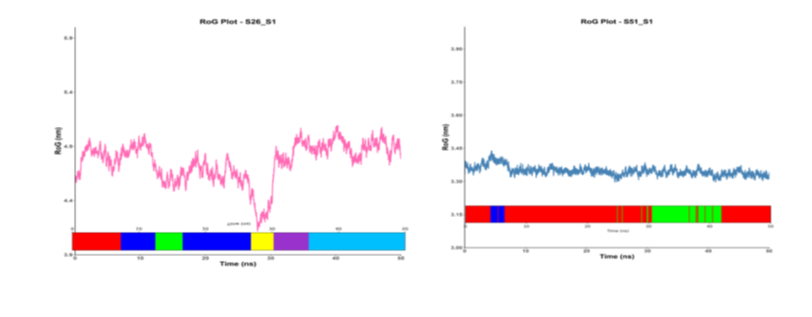


**D**

**E**
